# Factors Associated with Sexual Risks and Risk of STIs, HIV and Other Blood-Borne Viruses Among Women Using Heroin and Other Drugs: A Systematic Literature Review

**DOI:** 10.1007/s10461-018-2238-7

**Published:** 2018-08-02

**Authors:** L. Medina-Perucha, H. Family, J. Scott, S. Chapman, C. Dack

**Affiliations:** 1 Department of Pharmacy and Pharmacology, University of Bath, Bath, BA2 7AY UK; 2 Department of Psychology, University of Bath, Bath, UK; 3 5 West, 2.52, Department of Pharmacy and Pharmacology, University of Bath, Bath, BA2 7AY UK

**Keywords:** HIV/aids, Sexually transmitted infections, Women using heroin and other drugs, Sexual risks

## Abstract

This systematic literature review identified factors associated with sexual risks related to sexually transmitted infections (STI), HIV and other blood-borne viruses (BBV) among women using heroin and other drugs. The search strategy included five databases (PubMed, EMBASE, PsycNET, Web of Science, Scopus), and PsycEXTRA for grey literature. Out of the 12,135 publications screened, 30 peer-reviewed articles were included. Most publications were cross-sectional (n = 25), quantitative (n = 23) and included 11,305 women. Factors identified were: (1) socio-demographics; (2) gender roles and violence against women; (3) substance use; (4) transactional sex; (5) partner characteristics, partner’s drug use, and context of sex; (6) preferences, negotiation and availability of condoms; (7) HIV status and STIs; (8) number of sexual partners; (9) love and trust; (10) reproductive health and motherhood; and (11) risk awareness and perception of control. Overall, this review highlights important implications for future research and practice, and provides evidence for developing STI/BBV preventive strategies.

**Electronic supplementary material:**

The online version of this article (10.1007/s10461-018-2238-7) contains supplementary material, which is available to authorized users.

## Resumen

Esta revisión sistemática identifica factores asociados con el riesgo de enfermedades de transmisión sexual, VIH y otros virus de transmisión sanguínea, en mujeres que consumen heroína y otras drogas. La búsqueda bibliográfica se realizó en cinco bases de datos (PubMed, EMBASE, PsycNET, Web of Science, Scopus), y en PsycEXTRA para literatura gris. De las 12,135 publicaciones revisadas, 30 artículos publicados en revistas científicas se incluyeron en esta revisión sistemática. La mayoría de publicaciones son transversales (n=25), cuantitativas (n=23), e incluyen 11,305 mujeres. Los factores identificados son: 1) características sociodemográficas; 2) roles de género y violencia de género; 3) uso de sustancias; 4) trabajo sexual; 5) características y consumo de sustancias de la pareja sexual, y contexto de las relaciones sexuales; 6) preferencias, negociación y disponibilidad de preservativos; 7) estatus de VIH y otras enfermedades de transmisión sexual; 8) número de parejas sexuales; 9) amor y confianza; 10) salud reproductiva y maternidad; y 11) concienciación del riesgo y percepción de control. Esta revisión sistemática presenta importantes implicaciones para la investigación y prácticas preventivas. Además, reúne evidencia para el desarrollo de campañas para la prevención de enfermedades de transmisión sexual, VIH y otros virus de transmisión sanguínea.

## Introduction

Women using heroin and other drugs (WHOD) are particularly vulnerable to sexually transmitted infections (STIs), HIV and other blood-borne viruses (BBVs) [1–13]. Sexual risk practices (e.g., condomless sex) and experiencing violence have been suggested to contribute to this increased vulnerability [2, 3, 13–21]. These sexual risks are more prevalent among women because of gender inequities and gender-based violence towards women [7, 13, 22–24]. Women are also at higher risk due to their higher engagement in transactional sex [25–28]. Women are also more commonly affected by asymptomatic STIs [9], which may lead to delays in help-seeking behaviours and, therefore, timely screening, diagnosis and treatment.

The factors associated with the increased vulnerability to sexual risks account for the psychological, social, cultural, economic, organisational and political elements that are linked to sexual health. Contrarily to *determinants* of health, factors do not infer causality [29–32]. As there are also factors that may promote health behaviour change, it is crucial to understand the interplay of factors that have a role in the heightened vulnerability to sexual risks among WHOD, and the sexual transmission of STIs, HIV and other BBVs. Rather than merely focusing on changing drug use behaviour, it is also crucial to understand how we might change women’s vulnerability to sexual risks that are known to be associated with an increased risk of STIs, HIV and BBVs [9].

Preventive strategies for BBVs (and especially HIV) have been among the main public health priorities worldwide since the 1980s [33], when the first cases of HIV/AIDS were reported [34]. Most research and preventive programmes have focused on HIV transmission (and more recently viral hepatitis) via unsafe drug use (i.e., sharing needles and paraphernalia), overlooking sexual contact as a main vector of infection [18, 35–38]. Programmes tackling STIs have been predominantly focused on HIV prevention [36, 39, 40]. Since the Second World War, programmes tackling viral hepatitis have been associated with vaccines development and the discovery of new hepatitis viruses [41]. Recently, research has focused on responding to epidemics and outbreaks, recording prevalence rates, and developing preventive interventions, especially for HIV/AIDS and Hepatitis C. Even though it is crucial to recognise the importance of taking a social ecological approach to understand STI/BBV risk [13, 42–45], the evidence of the psychosocial and socio-structural factors associated with sexual risks remains scarce and unclear. This is due to the individualistic approach often taken in research and STI/BBV public health strategies for WHOD.

The main aim of this systematic literature review is to identify factors associated with sexual risks^1^ that lead to a heightened risk for STIs, HIV and other BBVs among WHOD. A secondary aim is to review the nature and quality of the evidence available. A critical approach is taken to highlight gaps in the evidence base and implications for the development of STI/BBV preventive strategies.

## Methods

### Eligibility Criteria

We included papers that identified factors relating to sexual risks, among adult (≥ 18 years) heterosexual women, or women who have sex with women, that were heroin or polydrug users whose primary drug of use was heroin. Studies included were of qualitative and quantitative methodologies. We excluded reviews and publications that were not in English language, studies focusing on the effectiveness of an intervention/treatment or where heroin was not the most prevalent drug of use, and studies where findings were not provided for female participants separately. The search was restricted to publications published between 1995 and end of June 2016. An inductive approach was taken for this review. All papers that discussed outcomes of sexual risks, including sexual experiences and sexual practices that may contribute to an increased exposure to STI/BBVs (e.g., experiencing sexual violence or selling sex) were included, regardless of how the outcomes/factors were measured or the time when they occurred.

### Search Strategy

**Fig. 1:**
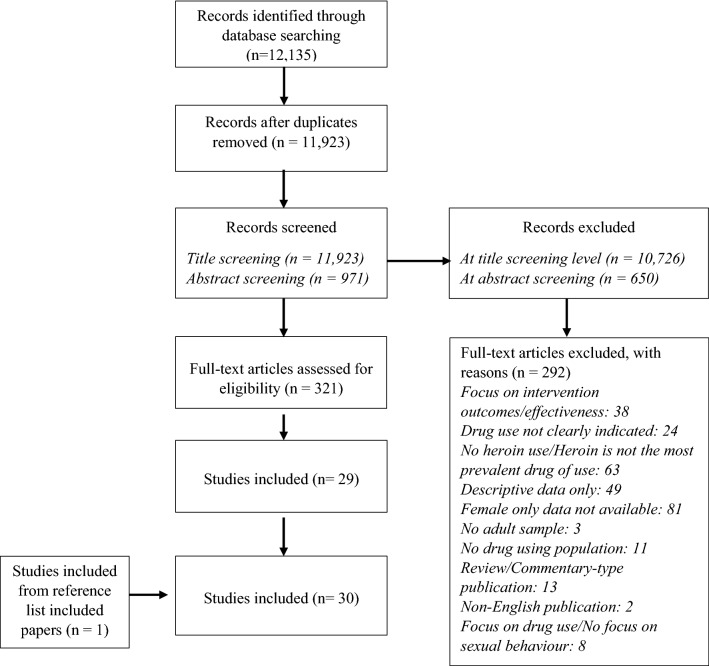
Flow diagram of study selection criteria

The search strategy included five databases: PubMed, EMBASE, PsycNET, Web of Science and Scopus. PsycEXTRA was used for grey literature and other publications. Study authors were contacted when there was no full-text access, and to identify potential additional studies. Search terms included were “women*”, “heroin use*”, “sexual behaviour*”, and “HIV” (see Fig. 1). The search and study selection were performed by the first author (LMP). The second (HF) and last (CD) authors reviewed ten percent of the publications at all screening stages. Three researchers (LMP, HF, and CD) met regularly to discuss each stage of the screening. Any disagreements regarding the inclusion or exclusion of papers were resolved over the meetings.

### Search Outcome

Thirty peer-reviewed articles were included in the review. Reasons for exclusion, ordered from most to least common, were: (1) women-only data were not available; (2) study participants did not use heroin, or heroin was not the most prevalent drug of use; (3) the paper provided descriptive data of sexual risks only, and did not relate these to psychosocial or socio-structural factors; (4) the focus of the paper was on intervention outcomes or intervention effectiveness; (5) drug use was not clearly indicated; (6) the paper was a review or a commentary-type publication; (7) study participants were from the general population rather than from drug-using populations; (8) the focus was on drug use, or there was no focus on sexual risks; (9) most study participants were below 18 years old; and (10) the publication was not in English (see Fig. 1).

### Quality Analyses

The PRISMA 2009 guidelines [46] and the Standard Quality Assessment Criteria for Evaluating Primary Research Papers from a Variety of Fields [47] were used for quality assessment purposes. Inter-rater reliability between the three reviewers was calculated at all screening stages (i.e., title, abstract, and full-text screening). Cohen’s Kappa was 0.5 on average, indicating a moderate and acceptable degree of agreement [48]. The inclusion/exclusion of papers was discussed between the three reviewers to reach full agreement. This systematic review was registered with PROSPERO (Reference PROSPERO 2016: CRD42016039842), available from http://www.crd.york.ac.uk/PROSPERO/display_record.asp?ID=CRD42016039842.

## Results

### Study Characteristics

A total of 30 peer-reviewed articles were included in this review. Most publications were cross-sectional (n = 25), four were longitudinal [49–52], and one was a case study [53]. There were 23 quantitative studies and seven qualitative studies [53–59]. The studies were conducted in several countries, but most commonly in the United States (n = 13). All papers were published between 1995 and 2015, and data was collected over a period between 2 weeks and 9 years. Seventeen publications were women-only studies [51, 52, 57, 58, 60–72]. See Table 1 for further details on the papers’ characteristics.

**Table 1:** Main characteristics of included papers

Reference	Country	Study type and setting	Sample	Data collected	Factors identified	Measures	Quality score
Gossop et al. [60]	United Kingdom	Quantitative; cross-sectional; community	N = 51 female sex workers	Socio-demographics; initiation into prostitution; current sexual behaviour with clients; use of condoms; drug injection behaviours; alcohol use; relationship between their drug use and prostitution; sexual behaviours with non-paying sexual partners; HIV status; number of partners and behaviours	Substance use (alcohol use, drug use); sex work; partner characteristics, partner’s drug using-patterns, and context of sex; HIV status and other STIs	Structured interviews; self-completion non-standardised questionnaire	4/16
Nyamathi et al. [61]	United States	Quantitative; cross-sectional; community	N = 378 impoverished women injecting drug users, or partners of injection drug users	Socio-demographics; sexual activity (unprotected sex in the last 6 months; unprotected sex with personal partners; number of persons they had sex with in that period); drug use; barriers to condom use; barriers to needle cleaning	Socio-demographics (age, ethnicity, housing conditions); substance use (drug use); partner characteristics, partner’s drug using-patterns, and context of sex; preferences, negotiation and availability of condoms; HIV status and other STIs; having multiple partners; reproductive health and motherhood	Drug use questionnaire (revised from the AIDS initial assessment questionnaire (AIA); 14-item condom use subset of the AIA (only women who had unprotected sex with their partners); 10-item needle cleaning subset of the AIA (only women who reported injection drug use)	5/16
Grella et al. [62]	United States	Quantitative; cross-sectional; community	N = 158 women in methadone maintenance treatment	Socio-demographics; polydrug use; alcohol use; depression; illegal activity; lives with drug-using partner; negative family history; sex abuse history; number of needle-sharing partners; sex behaviours; treatment retention	Socio-demographics (age, education); substance use (alcohol use, drug use); sex work; partner characteristics, partner’s drug using-patterns, and context of sex; having multiple partners	Face-to-face interviews (based on the natural history interview)	16/16
Carlson [54]	United States	Qualitative; cross-sectional; community	N = 62 injecting drug users (number of women not specified)	Socio-demographics; history of drug use; drug dealing; use of shooting galleries; needle transfer and circulation; needle cleaning; AIDS knowledge; sexuality; gender roles; condom use	Gender roles and violence against women; partner characteristics, partner’s drug using-patterns, and context of sex	In-depth qualitative interviews; participant observation	11/20
El-Bassel et al. [63]	United States	Quantitative; cross-sectional; community	N = 145 women in methadone clinics	Socio-demographics; drug use (previous 30 days, past year and lifetime); sexual behaviours (sexual activity and sex work past year); partner violence (physical, life-threatening, or sexual abuse by intimate partner); childhood sexual abuse; childhood physical abuse (occurrence, number of times, before age 16, that they suffered from physical abuse by parent or guardian); witnessing their mothers being abused by her intimate partner	Gender roles and violence against women	Drug use and drug risk behavior questionnaire; other measures not specified	16/16
Gilbert et al. [64]	United States	Quantitative; cross-sectional; community	N = 147 women in methadone maintenance treatment	Socio-demographics; drug use; sexual risk behaviour; childhood sexual abuse; childhood physical abuse; partner violence (physical abuse; life-threatening abuse; sexual abuse)	Gender roles and violence against women	Not specified	16/16
Tortu et al. [65]	United States	Quantitative; cross-sectional; community	N = 320 women	Socio-demographics; risk factors (e.g., injection drug use, HIV serostatus; non-commercial sexual events (incl. partner characteristics; relationship-specific and event-specific variables)	Substance use (alcohol use, drug use); partner characteristics, partner’s drug using-patterns, and context of sex; preferences, negotiation and availability of condoms; HIV status and other STIs; love and trust; reproductive health and motherhood; risk awareness and perception of control	Face-to-face structured interviews; biological testing for cocaine and morphine	15/16
Albertín-Carbó et al. [55]	Spain	Qualitative; cross-sectional; community	N = 113 (n = 36 women) on methadone treatment	Socio-demographics; daily life activities (including sexual behaviours)	Sex work; partner characteristics, partner’s drug using-patterns, and context of sex; reproductive health and motherhood	Ethnographic observation	15/20
Epele et al. [56]	United States	Qualitative; cross-sectional; community	N = 35 (n = 25 women) injecting drug users from Latino minorities	Socio-demographics; characteristic of gender relationships; drug history; current drug use; drug treatment history; injection behaviours; sexual behaviours (sex work; sexual behaviours in stable relationships)	Gender roles and violence against women; sex work	In-depth interviews	13/20
Hansen et al. [57]	Puerto Rico	Qualitative; cross-sectional; national	N = 311 female sex workers	Socio-demographics; specific sexual behaviours; how sexual behaviours are requested and negotiated; who determined the sexual behaviours; whether any behaviours were refused; where the behaviours were performed; how much time they spent with the client; how much and with what they were paid; how and where they were left after the encounter; what they did immediately after the encounter; what they did with the money earned; use of condoms and other forms of protection; how protection was discussed; who provided the protection	Gender roles and violence against women; sex work; partner characteristics, partner’s drug using-patterns, and context of sex; reproductive health and motherhood	Open-ended survey question: “Describe your experience with your last client from the time you encountered him until he left” (part of a 209-item survey instrument)	17/20
Miller et al. [73]	Canada	Quantitative; cross-sectional; community	N = 1437 (n = 597 women) injecting drug users	Socio-demographics; drug use and drug-using risk behaviours; sex work; sexual abuse; sexual history; condom use	HIV status and other STIs	Not specified	15/16
Miller et al. [74]	United States	Quantitative; cross-sectional; community	N = 257 (n = 92 women)	Socio-demographics; drug use; characteristics of network members; drug treatment; sexual behaviours in the past 30 days (not partner specific); characteristics of sexual partners	Socio-demographics (age); substance use (drug use); partner characteristics, partner’s drug using-patterns, and context of sex; HIV status and other STIs; having multiple partners	Structured face-to-face interviews	14/16
Sánchez et al. [76]	United States	Quantitative; cross-sectional; community	N = 241 (n = 84 women) heroin sniffers with no history of injection drug use	Socio-demographics; history drug use; drug use networks; sex history; criminal justice history	Substance use (drug use)	Modified AIDS risk questionnaire	9/16
Tyndall et al. [49]	Canada	Quantitative; longitudinal; community	N = 1400 (n = 505 women) injecting drug users	Socio-demographics; history of injection drug use in the preceding month; sexual behaviours; health services utilisation (e.g. emergency departments; clinics; hospitals)	Socio-demographics (ethnicity, housing conditions, legal involvement); substance use (drug use); sex work	Interview administered questionnaire	8/16
Gore-Felton et al. [66]	Russia	Quantitative; cross-sectional; community	N = 188 young drug users (18–25 years old)	Socio-demographics; history of STIs; injection drug use behaviour and drug-using behaviours; sexual behaviour	Socio-demographics (age); substance use (drug use)	Non-validated 72-item survey assessment	4/16
Medrano et al. [75]	United States	Quantitative; cross-sectional; community	N = 696 (n = 358 women) injecting drug users	Socio-demographics; substance use histories; sexual risk behaviours; histories of sexually transmitted diseases; childhood trauma (physical; sexual; emotional abuse; physical; emotional neglect)	Socio-demographics (age, education, marital status); gender roles and violence against women; substance use (drug use)	Pre-assessment with the risk behavior assessment (RBA); childhood trauma questionnaire (CTQ)	15/16
Wang et al. [67]	China	Quantitative; cross-sectional; community	N = 171 female sex workers	Socio-demographics; sexual behaviours; drug-using behaviours; HIV knowledge and attitudes	Socio-demographics (marital status, employment and financial aspects); substance use (drug use); sex work	77-item self-reported questionnaire	9/16
Bell et al. [68]	United States	Quantitative; cross-sectional; community	N = 251 women injecting/non-injecting drug users	Socio-demographics; drug-using patterns; sexual behaviours (incl. age at sexual debut; lifetime and current sexual history; STI history; frequency of unprotected and protected sex with steady, casual; sex trade partners); HIV and Hepatitis C screening and post-test counselling provided	Socio-demographics (sexual orientation)	Non-validated questionnaire	16/16
Lee et al. [58]	Taiwan	Qualitative; cross-sectional; community	N = 40 women injecting drug users in prison	Socio-demographics; acceptability and personal evaluation of health education materials/strategies; perceptions and personal evaluation of prison-based health education for female drug users; knowledge and health beliefs of the sample relating to HIV/AIDS; relationships between HIV/AIDS and drug use; issues relating to HIV testing resources; HIV/AIDS preventive behaviours and strategies; HIV/AIDS issues specific to women (e.g. mother-to-child transmission through breast feeding)	Sex work; partner characteristics, partner’s drug using-patterns, and context of sex; love and trust	Focus groups	18/20
Gu et al. [51]	China	Quantitative; longitudinal; community	N = 281 female sex workers and injecting drug users	Socio-demographics; perceived economic pressure; severity of drug dependence; questions on HIV-related risk behaviours	Socio-demographics (age, employment and financial aspects); substance use (drug use); sex work; HIV status and other STIs	Pre-tested non-validated questionnaire; 11-item revised Chinese Opiate Additive Severity Index-Revised (OASI-R)	15/16
Lam [59]	Vietnam	Qualitative; cross-sectional; community	N = 75 (n = 31 women) injecting drug users	Socio-demographics; sexual relations and risk behaviours; drug use; social contexts of multiple sexual relations; risk-taking behaviours; Argot/slang used by members of IDUs’ networks; social context; daily life activities	Gender roles and violence against women; sex work; partner characteristics, partner’s drug using-patterns, and context of sex; love and trust	Focused ethnographic interviews; focus groups; participant observation; case study research	15/20
Gu et al. [78]	China	Quantitative; cross-sectional; community	N = 162 (n = 59 women) injecting drug users	Socio-demographics; self-reported HIV status; perceived family relationship; perceived social isolation; drug-using patterns; needle sharing; sex work; sexual history; condom use	Substance use (drug use)	Not specified	16/16
Cavanaugh et al. [69]	United States	Quantitative; cross-sectional; community	N = 214 black and white women	Socio-demographics; drug use; sexual behaviour; history of STIs; HIV status	Socio-demographics (ethnicity)	The HIV risk behavior interview	16/16
Peng et al. [70]	Taiwan	Quantitative; cross-sectional; national	N = 263 HIV ± women in prison	Socio-demographics; drug-using risk behaviours; sexual-related risk behaviours; social factors (having drug-using sexual partner within 6 months prior to incarceration; working in nightclubs or bars; experience of physical abuse; exchanging sex for money or drugs)	HIV status and other STIs	Non-validated questionnaire	9/16
Gaines et al. [52]	Mexico	Quantitative; longitudinal; community	N = 584 (baseline); N = 567 (follow-up) female sex workers	Socio-demographics; condom use; drug-using risk behaviours; sexual risk behaviours (incl. history of STIs); sex working location; HIV and STI testing was provided	Socio-demographics (age, education, marital status, employment and financial aspects); substance use (alcohol use, drug use); sex work; preferences, negotiation and availability of condoms	Face-to-face interviews; biological testing for HIV/STIs	15/16
Goldenberg et al. [71]	Mexico	Quantitative; cross-sectional; national	N = 214 female sex workers	Socio-demographics; drug use; involuntary sex exchange; client interactions; intimate partner violence; social-structural factors; work environment; gender-based violence; HIV/STI testing	Socio-demographics (age, ethnicity); gender roles and violence against women; substance use (drug use); sex work; partner characteristics, partner’s drug using-patterns, and context of sex; preferences, negotiation and availability of condoms; HIV status and other STIs	Non-validated questionnaire; blood specimens (for HIV/STI testing)	15/16
Mackesy-Amiti et al. [50]	United States	Quantitative; longitudinal; community	N = 561 (n = 209 women) non-injecting drug users	Socio-demographics; alcohol use; injection and non-injection drug use; sexual activity	Socio-demographics (age, education, ethnicity, housing conditions); substance use (drug use); partner characteristics, partner’s drug using-patterns, and context of sex	Audio-computer-assisted self-interview (ACASI)	15/16
Iversen et al. [72]	Australia	Quantitative; cross-sectional; national	N = 5378 women injecting drug users	Socio-demographics; drug use/history; sexual risk behaviours in the preceding month; HIV and HCV testing; history of opioid substitution treatment	Socio-demographics (sexual orientation)	Non-validated questionnaire	14/16
Syvertsen et al. [77]	Mexico	Quantitative; cross-sectional; national	N = 214 couples (n = 214 female sex workers)	Socio-demographics; lifetime and recent sexual and drug-related HIV risk behaviours (unprotected sex; concurrent sexual partners; syringe sharing); depression; relationship-level variables (relationship satisfaction; prevalence of past-year verbal and physical conflict); emotional constructs of love and trust	Love and trust	Computerised non-validated questionnaires; revised conflict tactics scale (subscales for psychological aggression, physical assault, injury or sexual assault); 19-item triangular love scale (adapted); 8-item dyadic trust scale (adapted)	16/16
Syvertsen et al. [53]	Mexico	Qualitative; case study	N = 2 (heterosexual couple); n = 1 female sex worker, injecting drug user	Socio-demographics; drug-using patterns; drug-using risks; romantic relationships; love and trust; sex work; partner characteristics; life story; sexual health risks	Sex work; love and trust	Ethnographic observation; field notes	14/20

All participants in the included studies were using drugs

The quality of the quantitative studies ranged between 4 and 16, and the average score was 12.7 (0 being the minimum and 16 the maximum possible score). The quality scores for the qualitative studies ranged between 11 and 18, and the average was 14.7 (0 being the minimum and 20 the maximum possible score) (see Supplementary Material). Only six papers included information about risk bias assessment [51, 59, 62, 67, 70, 73]. Strategies included training, regular meetings, participant checks, reflexive analysis, inter-rater checks, and assurance of qualitative data saturation. Few studies used specific theoretical approaches [53, 55, 56, 59, 62, 74]. None of the publications were excluded based on their quality, to capture the nature and quality of the evidence available in the area of study covered in this review. However, the range in the papers’ quality should be taken into account when interpreting the findings of this review.

### Sample Characteristics

There was a significant amount of missing and heterogeneous data within the included papers. The sample characteristics presented are based on the available data only. The review included 11,305 women based on all papers but one [54] in which the number of female participants was not specified. The mean age was 31 years (*SD *= 5.11) [49, 53, 56, 57, 59–64, 66–71, 74, 75], 25.5% were African American, 22.5% Latin/Hispanic, 18.6% Indigenous Australian, 15.4% White/Caucasian, 11.6% Asian, 4.9% Indigenous (North American), and 1.5% were from non-specified ethnicities [49, 51, 56, 60–63, 65, 67–69, 71, 72, 74, 75]. Heroin was used by 44.6% of women, followed by methadone (22.3%), cocaine and/or crack cocaine (21.7%), methamphetamine (16.8%), alcohol (7.6%), speedball (3.2%), cannabis (2.3%), tobacco (1%), liquefied opium/opium (0.2%), inhalants (0.2%), and heroin together with other narcotics (0.2%) [49, 51–53, 55–73, 75–77]. Seven papers included data on sexual orientation [52, 56, 58, 68, 70, 74, 75], and indicated that 78.3% women in these studies were heterosexual and 21.7% were lesbian or bisexual. Data available revealed that 47.8% women were married, in common-law or cohabiting, 25% were single, 24% were separated, divorced or widowed, and 3.2% had a non-specified marital status [49, 51, 52, 56, 57, 60–65, 67, 70, 71, 74, 75, 77, 78]. Over a third of women (34.9%) had been homeless at some point in the last year, and 41.5% had been homeless at some point in their lives [60, 62, 63, 65, 68, 69, 74]. More than half (58.2%) had been in prison [58–60]. Most women engaged in transactional sex^2^ at the time they participated in the studies (89.6%), 6.1% at some point in the previous year, and 4.3% had exchanged sex for money and/or drugs at some point in their lives [49–53, 57, 63, 64, 67–74, 76–78].

### Synthesis of Results

The inductive nature of this study led to identifying outcomes a posteriori, so as a result of the data analysis. There were six main outcomes in the included papers that were found to be linked to STI/BBV risk. These were (1) condom use; (2) transactional sex; (3) experiencing sexual violence; (4) sexual activity; (5) type and characteristics of the sexual partner; and (6) drug use with sex partners.

Eleven factors were identified to be associated with the sexual risk outcomes above, and ultimately with STI/BBV risk. These were (1) socio-demographics; (2) gender roles and gender-based violence; (3) substance use; (4) transactional sex; (5) partner characteristics, partner’s drug-using patterns, and context of sex; (6) preferences, negotiation and availability of condoms; (7) HIV status and sexually transmitted infections; (8) number of sexual partners; (9) love and trust; (10) reproductive health and motherhood; and (11) risk awareness and perception of control.

Some sexual risk outcomes were also found to be factors associated with sexual risks (e.g., transactional sex was a factor found to be associated with condom use). The identification of factors and sexual risk outcomes was based on the conceptualisation made in each of the papers included in the review. This reflects both the lack of homogeneity of the evidence available, and the complexity of interrelations between outcomes and factors of STI/BBV risk.

This section has been organised by sexual risk outcomes. An explanation on the evidence of the relationship between research outcomes and each of the identified factors is provided (see Table 2).

**Table 2:** Identified factors and outcomes

Factors	Outcomes	Reference included papers
*Socio-demographics*		
Age	Condom use	[50–52, 61, 62, 66, 71, 74]
	Transactional sex	[50, 75]
	Number of sexual partners	[66]
Education	Condom use	[52, 62]
	Transactional sex	[62, 75]
Ethnicity and country of origin	Condom use	[50, 61, 71]
	Transactional sex	[49, 50, 69]
	Number of sexual partners	[61]
	Type and characteristics of the sexual partner	[50, 69]
	Sexual violence	[71]
	Sexual activity	[69]
	Drug use with sexual partners	[69]
Sexual orientation	Transactional sex	[68, 72]
	Number of sexual partners	[68]
	Sexual activity	[68]
	Type and characteristics of the sexual partner	[68, 72]
Marital status	Condom use	[52]
	Transactional sex	[67, 75]
Housing conditions	Condom use	[50, 61]
	Transactional sex	[49]
	Number of sexual partners	[50]
	Sexual activity	[50]
Employment and financial aspects	Condom use	[51, 52]
	Transactional sex	[51, 67]
Legal involvement	Transactional sex	[49]
*Gender roles and violence against women*	Condom use	[56, 59, 64, 71]
	Transactional sex	[54, 56, 63, 64, 75]
	Number of sexual partners	[64]
	Type and characteristics of the sexual partner	[64]
	Sexual violence	[56, 59, 71]
*Substance use*		
Alcohol use	Condom use	[52, 60, 62, 65]
Drug use	Condom use	[51, 52, 60–62, 65, 74]
	Transactional sex	[49–51, 53, 56, 57, 59, 60, 66, 67, 75, 76, 78]
	Number of sexual partners	[50, 66, 76]
	Type and characteristics of the sexual partner	[76]
	Sexual violence	[71]
	Sexual activity	[60]
*Transactional sex*	Condom use	[51, 52, 55–60, 67]
	Number of sexual partners	[51, 62, 67]
	Sexual violence	[54, 56, 57, 59, 71]
	Sexual activity	[67]
	Type and characteristics of the sexual partner	[49, 67]
*Partner characteristics, partner’s drug use, and context of sex*	Condom use	[50, 54, 55, 57–62, 65, 71, 74]
	Sexual violence	[71]
*Preferences, negotiation and availability of condoms*	Condom use	[52, 61, 65, 71]
*HIV status and sexually transmitted infections*	Condom use	[51, 61, 65, 70, 73, 74]
	Transactional sex	[51, 73]
	Number of sexual partners	[73]
	Sexual violence	[71]
	Type and characteristics of the sexual partner	[70, 73]
*Number of sexual partners*	Condom use	[61, 62, 74]
	Type and characteristics of the sexual partner	[62]
*Love and trust*	Condom use	[53, 58, 59, 65, 77]
*Reproductive health and motherhood*	Condom use	[55, 57, 61, 65]
*Risk awareness and perception of control*	Condom use	[65]

## Condom Use

Factors identified to be correlated to condom use were (1) socio-demographics; (2) gender roles and gender-based violence; (3) substance use; (4) transactional sex; (5) partner characteristics, partner’s drug-using patterns and context of sex; (6) preferences, negotiation and availability of condoms; (7) HIV status and sexually transmitted infections; (8) number of sexual partners; (9) love and trust; (10) reproductive health and motherhood; and (11) risk awareness and perception of control.

### Socio-demographics

*Age* There seemed to be a relationship between age and condom use. Six papers found that age was significantly correlated with engaging in condomless sex [50, 51, 61, 62, 66, 74]. However, data from another paper indicated that this correlation was non-significant [52]. The nature of the association between age and condom use was unclear. In one study, women over 35 years old were significantly more likely to have condomless sex, compared to younger women [61], whereas condom use was marginally and positively associated with age in another study [74]. Among women who engaged in transactional sex, those who were 36 years old or older were more likely to use condoms inconsistently^3^ followed by women between 26 and 30, women between 31 and 35, and 25-year-old women and younger [51].

*Education* Data on formal education and condom use were conflicting. Evidence from one paper indicated that graduating from high school was negatively correlated to condom use [62]. Education attainment was found to be non-significantly correlated to using condoms in another study [52].

*Ethnicity* There were differences in condom use and reported barriers for condom use among women from different ethnicities. White women were more likely to engage in condomless sex with a main partner than Black women [50]. Compared to African Americans, Latina women were more likely to report partner’s dislike of condoms as a barrier for condom use. In turn, African American women reported greater lack of skills using and negotiating condom use, difficulties to get condoms, and discomfort using condoms [61]. Compared to Latinas, there were more reports of African American women not considering using condoms when they were under the influence of drugs [61]. African Americans were also more likely to believe that their partner did not have AIDS, and that they could not transmit HIV to their partners compared to Latinas [61]. These beliefs were associated with having condomless sex.

*Marital Status* Only one study reported a relationship between marital status and condom use. The findings from this study indicated that there was a non-significant positive correlation [52].

*Housing Conditions* Higher reports of condomless sex were made by homeless women who injected drugs, compared to those in drug recovery programmes [61]. Women who were cohabiting were more likely, than those who were not, to have condomless sex with steady partners,^4^ and reported more condomless anal sex [50].

*Employment and Financial Aspects* Transactional sex was associated with an increased likelihood to use condoms inconsistently in the previous 6 months if women perceived great economic pressure due to drug using practices and living expenses [51, 52]. There was also a direct impact of economic pressure on general HIV-related sexual risk and on inconsistent condom use in the past 6 months [51]. One study suggested a non-significant correlation between self-rated financial situation and consistent condom use^5^ [52].

### Risk Awareness and Perception of Control

Condom use was predicted when women perceived control over condom use, and using a condom made women feel responsible [65]. A frequent barrier to condom use was the belief that women did not need protection for sex [65].

### Reproductive Health and Motherhood

The fertility of WHOD was perceived to be significantly reduced as a result of their heroin use. The use of condoms was dependent on whether women wanted to have a child or prevent pregnancy. As expected, condomless sex was common among women who wanted to become pregnant [55], had a partner that wanted a child [61], or had a tubal ligation [65]. On the contrary, condom use was higher among those women who wanted to prevent pregnancy [61, 65]. On the other hand, condomless sex was frequent among mothers involved in transactional sex. This was to maintain regular clients and earn more money to provide for their children [57].

### Number of Sexual Partners

The number of male sexual partners was positively correlated with a lack of condom use [62]. These differences were however not significant over a 3-year period [61]. Having had two or more sexual partners in the last 30 days was negatively correlated with the risk of having condomless sex [74]. When adjusting for other variables, having more than two sexual partners was not significantly associated with condomless sex [74].

### Love and Trust

In romantic relationships, condomless sex was habitual as it was positively associated with feelings of love [77] and trust [58, 59]. Women expressed that suggesting condom use in steady relationships could raise concerns of infidelity and suspicion [58, 59], and that condoms created an emotional barrier with their partners [58]. However, although non-significant and inconsistent to the relationship between love and condomless sex, another study found that a predictor of condom use was women’s perception of closeness to the partner [65]. Yet, when considering the risk of infection, women had a sense of shared destiny and fatalism. Trust and love in romantic relationships translated into inconsistent condom use and an increased sense of security as women only had condomless sex with their partner. The likelihood of transmission was often perceived as unavoidable as part of their relationship and drug-using lifestyle [59]. Embarrassment talking about sex and difficulties negotiating condom use with intimate partners also hindered condom use [58].

Among women engaging in transactional sex, condoms were used to emotionally differentiate sexual encounters with clients and non-clients. Whereas condoms were used with clients, condomless sex was only reserved for romantic relationships [53]. In fact, love and trust were negatively correlated with never or rarely using condoms with clients in the previous month [77].

### Gender Roles and Gender-Based Violence

Most women reported having experienced physical and sexual violence by men, which significantly increased sexual risks such as having condomless sex [56, 64]. Physical violence was often interlinked with sexual violence, and sexual violence most commonly involved condomless sex. Surprisingly, another study found a non-significant relationship between condom use and experiencing sexual violence [71]. Gender-based violence was rooted in gender roles and power dynamics between men and women, in which men were dominant over women. Some women were opposed to losing power and taking a submissive role in relation to men [56, 59]. One strategy used by some women as a way to overcome their vulnerable position was to use condoms with casual and steady sexual partners [59].

### Substance Use

*Alcohol Use* Using alcohol was associated with a decreased frequency in condom use [62, 65]. The evidence regarding the impact of alcohol use before sex among women in transactional sex was contradictory. Two studies suggested that alcohol use before sex was not found to be a predictor of condom use [52, 60]. However, the findings from another paper indicated that alcohol use before sex and weekly alcohol consumption were significantly and negatively associated with consistent condom use with both regular and casual clients [52]. The frequency and quantity of alcohol use were significantly and positively associated with using condoms after using drugs [60]. This finding contrasts with another study, in which weekly alcohol use was found to be significantly and negatively associated with condom use with transactional sex clients [52].

*Drug Use* Condom use was found to be marginally and positively associated with polydrug use [62]. Condomless sex was more likely among women who used heroin with sexual partners [74]. A higher number of needle-sharing partners was related to condomless sex [62].

Transactional sex appeared to be associated with a decreased likelihood of condom use when using drugs [51, 52, 65]. Among transactional sex workers, drug use before sex and daily injecting were negatively associated with consistent condom use [52]. Longer duration and higher severity of drug use were positively correlated with inconsistent condom use and general HIV-related risk [51]. Another study indicated that self-reported severity of heroin and cocaine dependence, and the use of condoms for vaginal sex with transactional sex clients were not significantly correlated [60]. There was also a non-significant association between typical doses of heroin and cocaine, and condom use with clients, nor between typical doses and transactional sex participation [60]. In addition, no association was found between frequency (days per week) of heroin or cocaine use and condom use with clients [60].

### Transactional Sex

There were no significant differences in condom use between women engaging in transactional sex and those who did not [67]. Some women were unwilling to have condomless sex with clients for more money [60]. Other women involved in transactional sex reported to be in a more vulnerable position to refuse condomless sex with clients [56, 57, 59]. In some cases, transactional sex workers engaged in condomless sex to avoid losing clients over other transactional sex workers. Women were usually offered larger amounts of money to have condomless sex with clients, which could result in a higher likelihood of having sex without using condoms [55]. Also, even though women selling sex generally had to negotiate condom use as part of the exchange [57], some women resisted carrying condoms as they were afraid of being identified as transactional sex workers by the police and have legal problems [58]. Women working in indoor venues (i.e., bar, hotel or brothel) were significantly more likely to use condoms consistently with both regular and casual transactional sex clients, compared with women working outdoors (i.e., street, clients’ vehicles, shooting galleries, other public spaces) or in low-price guest houses [51, 52]. Positive interactions with clients facilitated condom use [57], although some women did not use condoms with trusted regular clients [57].

### Partner Characteristics, Partner’s Drug Using Patterns, and Context of Sex

Several barriers to condom use were related to characteristics of sexual partners. Known health status, personal characteristics (e.g., marital status), physical appearance (i.e., age, attractiveness and apparent hygiene), and sexual history were associated with condom use and seemed to lead women to refuse certain sexual acts such as anal or oral sex with non-clients [54, 55] and clients of transactional sex [57]. Women expressed how some men insisted on having condomless sex as they felt that condoms reduced sexual pleasure and were inconvenient [58]. Moreover, it was more unlikely for women to use condoms when the sexual partner had similar health conditions to them, denied being HIV positive or when women perceived partners as ‘similar to me’ [55].

Self-reported condom use with transactional sex clients was higher when women had vaginal and anal sex, and less likely for oral sex and masturbation [60]. Condom use was also found to be more likely when the partner or woman performed oral sex and, although non-significant, sex occurred at the woman’s home or on a special occasion (e.g., birthday, anniversary) [65]. Other predictors of condom use were having had sex with the same sexual partner in the past, length of time women knew the partner, and having sex with a steady or casual partner (only with a steady partner in multiple regression analyses) [65]. However, most women reported to ‘never’ use condoms with non-clients in another study [60]. Condom use was also hindered when condoms were unavailable [55]. Besides, there was a non-significant relationship between condom use and receiving social support from a sex partner [74].

Condom use and having needle-sharing partners were positively associated [62]. There was no significant association between having transactional sex clients who injected drugs and condom use [71]. However, condomless sex was associated with having sex with men who injected drugs, when these were not transactional sex clients [50, 61, 62]. In romantic relationships formed by a person who injects drugs (PWID) and a person who does not inject drugs (PWNID), condoms were generally used if the PWID in the relationship was HIV positive and the PWNID was HIV negative [55]. In sexual relationships between a PWID and a person who does not use drugs, knowing the partner’s drug using practices was key for HIV infection. When non-using women were not aware of the partner’s drug using practices, they seemed to be more likely to have condomless sex. However, HIV risk concerns increased when women knew that their partner was injecting drugs. In order to avoid partner’s concerns and continue having condomless sex, some drug users hid their drug using practices and health status, which increasingly heightened the risk of infection [59]. On the other hand, disparities on sexual desire were common in PWID-PWNID relationships. In this context, the increased sexual desire of the PWNID led to the rejection of condom use, as a way for the PWID partner (most often men) to please their partner [59].

### Preferences, Negotiation and Availability of Condoms

Condom use was facilitated when it was discussed, and especially when women were more willing to use condoms. Also, when both partners agreed on either using condoms or the partner insisted on using condoms [65]. Lack of skills using and negotiating condom use, dislike of condoms, discomfort [61, 65] and loss of pleasure using condoms were common reported barriers to condom use [65]. Other barriers were that using condoms made sex less intimate, either women or their partners did not feel like using condoms, a partner got angry about using condoms, and when the partner refused to use them [65]. Another study however found a non-significant relationship between condomless sex and having a partner insisting on not using condoms [71]. Condom use was also hindered when partners agreed on not using condoms, sex was unplanned, women could not afford to buy condoms [65], and condoms were unavailable [55, 61, 65]. However, another study suggested that there was no significant association between access to free condoms and consistent condom use [52]. On the other hand, some women expressed discomfort when negotiating condom use. This discomfort appeared to be caused by the fear of offending their partners when suggesting using condoms, and being afraid of getting hurt. These difficulties in negotiating condom use were a barrier to having sex with a condom [61].

## Transactional Sex

Factors associated with selling sex were socio-demographics, gender roles and gender-based violence, and substance use.

### Socio-demographics

*Age* There was conflicting evidence regarding the relationship between age and transactional sex. Whereas one paper indicated that these were correlated [50], another suggested a non-significant correlation [75].

*Education* As for age, there was contradictory evidence on the relationship between education and transactional sex. Graduating from high school was found to be negatively correlated to transactional sex [62]. This relationship was however found non-significant in another paper [75].

*Ethnicity* The likelihood of engaging in transactional sex was higher among Black women, compared to White, Hispanic and women from other ethnicities [50]. In another study, ethnicity was not found to be significantly associated with transactional sex, even though Indigenous Canadian women were less involved in transactional sex [49]. Compared to Black women, White women had more transactional sex clients [69]. Black women were, on average, older the last time they sold sex [69].

*Sexual Orientation* Both bisexual and lesbian women were more likely to engage in transactional sex, compared to heterosexual women [72]. The engagement in transactional sex was also higher among women who currently had sex with women (CSW), followed by women who had past sexual experiences with women (PSW) and women who never had sex with women (NSW) [68].

*Marital Status* Single women were more likely to have sold sex, compared to married women. This was significant when looking at the impact of emotional neglect, emotional abuse and physical neglect on transactional sex practices [75]. Extramarital sex was more likely among married women who were selling sex, compared to married women who did not engage in transactional sex [67].

*Housing Conditions* Being in unstable housing was more prevalent among women involved in transactional sex, who also lived in more deprived urban areas [49].

*Employment and Financial Aspects* Women who engage in transactional sex were more likely to have another job and were considered to be unemployed [67]. Although non-significant, there were associations between having two or more daily clients in the previous 2 weeks, and perceiving economic pressure due to being in debt, the need to support family members and drug use [51].

*Legal Involvement* A significant relationship was found between having been in jail in the previous 6 months and transactional sex [49].

### Gender Roles and Gender-Based Violence

Socially constructed gender roles and power dynamics in which men are dominant over women were associated with engaging in transactional sex [56]. In order to avoid assuming a submissive role in relation to men, some women obtained drugs and supported themselves through transactional sex [56].

On the other hand, experiencing physical and/or sexual violence was positively associated with engaging in transactional sex [54, 63, 64]. Physical and sexual violence were associated with having had a HIV-positive partner in the previous 30 days [64]. Specifically, childhood abuse was significantly correlated with transactional sex [64]. Another study found that childhood physical and sexual abuse did not increase the likelihood of being involved in transactional sex as an adult among Black women [75]. Only the severity of emotional and physical neglect, and emotional abuse were associated with an increased likelihood of transactional sex among Black women [75]. No form of abuse was significantly associated with transactional sex among White or Hispanic women [75].

### Substance Use

*Drug Use* Injecting drug use was associated with engaging in transactional sex [50, 66, 67, 76, 78]. In one of the studies, this relationship was found to be significant for non-White women only [50]. The primary drug of use was not a significant predictor for transactional sex practices [75]. Data from one of the included studies indicated that severity of drug dependence was significantly related to having two or more transactional sex partners in the week before taking part in the study [51]. It is important to highlight that no difference was found in this study between self-reported severity of dependence between women who did and did not engage in transactional sex [60]. Other studies found that heroin use was marginally higher among women engaging in transactional sex, although cocaine and crack use was more frequent among women involved in transactional sex [49]. Women who sold sex had been using drugs for a longer time, compared to women who did not engage in transactional sex [66]. Nonetheless, these two studies [49, 66] did not find significant associations between substance use and sexual practices. Sex was generally exchanged for money or drugs and, in some cases, to sustain the partner’s drug habits [53, 56, 59]. Sex-for-drugs exchanges were more common when women experienced withdrawal symptoms, as transactional sex was an accessible drug-seeking behaviour. Men often took advantage of women’s addiction and offered drugs in exchange for sex [56, 57, 59]. Women were also less selective with clients when they experienced withdrawal symptoms [60]. Having withdrawal symptoms was correlated with self-reported severity of dependence upon heroin [60]. Women would often use drugs before transactional sex as a coping mechanism and emotional barrier towards transactional sex. Using drugs before transactional sex increased women’s sexual health risks [56, 57, 59].

## Sexual Violence

Factors associated with experiencing sexual violence were socio-demographics, gender roles and gender-based violence, substance use, transactional sex and partner characteristics, partner’s drug using patterns and context of sex.

### Socio-demographics

*Ethnicity and Country of Origin* Among women engaging in transactional sex in Mexico, those born in the US who spoke English were more likely to report sexual violence [71].

### Gender Roles and Gender-Based Violence

The high threat of violence led women to take a subordinate role and to rely on men for protection from violence [56]. Women reaching for protection constructed relationships with men based on exchanging resources [56]. In the context of these relationships, the role of women was to attend living and drug use expenses [56, 59], whereas men were expected to offer safety. However, men did not always provide women with protection and were often abusive towards women themselves. Conflicts with partners were associated with drug distribution and with men’s sexual difficulties [56]. Women often felt ‘*used for sex*’ and stigmatised by men as, because of their drug use, women were regarded as ‘*easy’* and worthless [59]. Women then reported that they became even more vulnerable to sexual and physical violence and exploitation, from both their partners and other men [56, 59]. Those who were involved in transactional sex often reported having partners insisting on having sex or condomless sex, which was associated with sexual violence [71]. Among women engaging in transactional sex, those experiencing sexual violence were more likely to have a history of rape [71].

### Substance Use

*Drug Use* Women involved in transactional sex were more likely to experience sexual violence when they used drugs with clients [71].

### Transactional Sex

Selling sex made women particularly vulnerable to sexual and physical violence, and consequently increased sexual health risks [54, 56, 57, 59]. In this context, women’s exposure to violence [56, 57] and sexual health risks was particularly heightened [59]. Despite women’s high risk for HIV, sexual health concerns were less of a priority compared to other dangers of the ‘fear culture’ in which women lived [56]. Even though some women engaged in protective strategies (e.g., having regular clients, offering oral sex rather than vaginal/anal sex, resorting to stealing, working legally, and sometimes relying on welfare) to decrease these risks [56, 57], they were still the target of violent assaults [56]. Those women who reported cases of gender-based violence were disregarded by the police, which contributed to women’s feelings of powerlessness and the perpetuation of the constant threat of violence [56], and consequent sexual health risks.

Women involved in transactional sex usually experienced very poor and unsafe working conditions, which made it difficult for women to be selective with clients, to maintain good hygiene, and to avoid coercive encounters with clients [55]. Transactional sex in hotels or motels, living and working in the same location, and reports of bad/extremely bad working conditions were associated with an increased likelihood of reporting sexual violence [71]. The association between location and working conditions were non-significant in multivariate statistical analyses [71]. Women who had their transactional sex earnings administered by a partner, and those having to pay a manager or a pimp were more likely to have experienced sexual violence [71]. The relationship between sexual violence and having to pay managers or pimps was however not significant [71]. Also, the risk for HIV was related to the position of women engaging in transactional sex in the street hierarchy, where women working for a pimp were the most vulnerable as they usually worked in exploitative conditions [56]. Although some interactions with clients were positive, others turned out to be violent and coercive. In order to prevent violent situations, women involved in transactional sex preferred working with regular clients [56, 57].

### Partner Characteristics, Partner’s Drug Using Patterns, and Context of Sex

Women engaged in transactional sex were more vulnerable to experiencing sexual violence when they had drug-using (PWID and PWNID) clients, and more non-regular clients [71].

## Sexual Activity

Sexual activity included the number of sexual partners, initiation of sex, and general frequency of sexual activity. There were various factors identified to be related to sexual activity. These were socio-demographics, gender roles and gender-based violence, substance use, and transactional sex.

### Socio-demographics

*Age* According to the data of one of the included papers [66], women who initiated sex at a younger age were more likely to have multiple sexual partners.

*Ethnicity* African American women were more likely to have multiple sexual partners, followed by acculturated Latinas, and compared to low acculturated Latinas [61]. Compared to Black women, White women had their first sexual encounter at a younger age [69].

*Sexual Orientation* CSW initiated sex at a younger age, had more than one male sexual partner in the previous 6 months, and reported having had sex daily in the past 6 months, compared to PSW and NSW [68]. Also, CSW were more likely to have vaginal sex more than once a week, oral sex with casual partners, and having had anal sex [68]. PSW reported to engage in oral sex more than CSW and NSW [68].

*Housing Conditions* Women who were cohabiting were less likely to have more than one sexual partner, compared to women who were not cohabiting [50]. Women who were cohabiting were more likely, than those who were not, to have anal sex [50].

### Gender Roles and Gender-Based Violence

No significant associations were found between recent partner violence and having had sex with more than one partner in the past year [64].

### Substance Use

*Drug Use* Having multiple sexual partners was significantly correlated with higher drug injecting [50, 66] and crack use [76]. The relationship between number of sexual partners and drug injecting was significant for non-White women only in one of the studies [50]. Moreover, women were more likely to engage in a wider variety of sexual practices with clients after taking heroin and cocaine [60].

### Transactional Sex

Overall, women who sold sex had more sexual partners in the previous year and in their lifetime, compared to women who did not engage in transactional sex [62, 67]. Women who worked in hotels and in saloons and massage parlours had more clients than those working in other settings [51]. Women involved in transactional sex were also more likely to have had their first sexual experience at a younger age [67]. Women engaging in transactional sex who experienced sexual violence were younger and had initiated transactional sex at a younger age, compared to those who did not report sexual violence. This relationship was however non-significant [71].

## Type and Characteristics of the Sexual Partner

There were very few studies and a lack of robust data on the factors correlated with the type and characteristics of a sexual partner. The factors identified were socio-demographics, transactional sex, number of sexual partners, gender roles and gender-based violence, and substance use.

### Socio-demographics

*Ethnicity* White women were more likely than Black women to have a sexual partner who injected drugs [50]. Although non-significant, White women were younger the last time they had a steady partner [69].

*Sexual Orientation* Heterosexual women reported less casual sex, compared to bisexual and lesbian women. Bisexual women were more likely to have recent casual sex, and lesbian women were less likely to have sex with steady partners, compared to heterosexual women [72]. CSW were less likely to have steady male partners [68], and more likely to have vaginal sex more than once a week, oral sex with casual partners, having had anal sex, and having had a sexual partner diagnosed with an STI. In contrast with CSW and NSW, PSW engaged in oral sex more than four times a week [68].

### Transactional Sex

Selling sex was associated with women being more likely to have sex with strangers or a friend, rather than with a boyfriend or husband [67]. They were also less likely to have a regular sexual partner [49].

### Number of Sexual Partners

The evidence available indicated that the number of male sexual partners was positively correlated with the number of needle-sharing partners [62].

### Gender Roles and Gender-Based Violence

There were no significant associations between recent partner violence, and having sex with a PWID, having sex with a partner who had sex with someone else in the past year, and having sex with a partner who had an STI in the past year [64].

### Substance Use

*Drug Use* There were very few and robust data on the relationship between drug use and the type and characteristics of sexual partners. The evidence available suggested that crack use and having a partner that is a PWID were not associated [76].

## Drug Use with Sexual Partners

Socio-demographics and gender roles and gender-based violence were associated with women using drugs with sexual partners.

### Socio-demographics

*Ethnicity* Compared to Black women, White women reported higher use of drugs before and after transactional sex, and higher use of injecting drugs with steady and casual partners [69]. There was a trend for Black women to use more non-injecting drugs when engaging in transactional sex [69].

### Gender Roles and Gender-Based Violence

A non-significant relationship was found between experiencing sexual violence and an increased likelihood of using drugs with clients, among women involved in transactional sex [71].

## Discussion

The main aim of this review was to identify the factors associated with sexual risks and risk of STIs and BBVs among WHOD. A secondary aim was to determine the nature and quality of the evidence available.

### Aim 1: Factors Associated with Sexual Risks

A wide range of factors, from socio-demographic characteristics to social contexts of violence and power dynamics between women and men, were found to be associated with sexual risks among WHOD. The interplay of these factors remains uncertain, and there were a number of studies presenting contradictory findings. This indicates that there is currently a lack of strong evidence on the links between most factors and sexual risks. This point is further discussed in Aim 2 of the Discussion section. The most salient factors and implications for future research and service development are discussed below.

#### Gender-Based Violence: Power Inequities and Human Rights

Despite the ambiguity of the findings, the evidence between experiencing violence and sexual risks was found to be fairly robust. A relationship emerged between violence and engaging in transactional sex, having condomless sex, and having high risk sexual partners. Consistent with previous research [80], gender-based violence (GBV) was related to gendered power dynamics in sexual relationships [81]. Men exerted power over women in order to obtain resources from them (i.e., money or drugs), and forced women into sex and transactional sex. Women, especially those involved in transactional sex, were also often exposed to random violent physical and sexual assaults. In fact, rates of interpersonal violence among drug users have been found to be between 50 and 70% [82, 83], with the severity of substance use associated with the severity of violence [84–87]. Women are particularly at risk of intimate partner violence [88], which is related to condomless sex [89] and higher prevalence of HIV infection [90]. Living in a context of abuse increases women’s susceptibility to violence, deterring women from prioritising their sexual health, and making it impossible for them to prevent violent assaults. Experiencing psychological and/or physical violence was found to be a barrier to condom use, as women became afraid and disempowered to negotiate condom use [88, 91]. Women feared violence if they suggested condom use. Also, in situations of sexual violence, condomless sex was generally imposed by the aggressor so women had neither control over the assault nor their sexual health. Most of the data available were related to physical and sexual violence, even though emotional violence might be more widespread and might also have a strong impact on women’s exposure to sexual risks throughout their lives.

Overall, it is crucial that GBV is understood in the context of culturally constructed gender roles and power inequities experienced by women in relation to men [92–96]. According to Heise’s ecological framework for violence against women [97], violence occurs and it is influenced by gendered factors across a social ecology at different levels (individual, interpersonal, community and societal) [93]. Structural violence should also be acknowledged, as GBV is embedded in social systems and institutions [92, 93]. However, most strategies to prevent gender-based violence have focused on individual behaviours and health outcomes, rather than the elimination of GBV as a violation of human rights rooted on unequal power dynamics. As previous research has suggested, there is the need to equate the power relations between women and men and promote community-level changes, shifts in public discourse, and to focus on shaping social norms across all social ecology levels (i.e., individual, social, institutional, cultural and political level) [93, 95, 96]. Future research should then account for the different realities and multidimensionality of GBV to comprehensibly understand how it impacts the sexual health and wellbeing of WHOD.

#### Transactional Sex: Social Neglect and Structural Violence

Contrary to what previous research has suggested [98], no clear relationship was found between condom use and transactional sex with either clients or non-clients in the quantitative studies. Qualitative data suggested that some women might agree on having condomless sex in exchange for larger amounts of money, and to avoid losing clients to other transactional sex workers. Violent and coercive interactions with clients were found to hinder negotiating condom use as women were often coerced or forced to have condomless sex. Transactional sex exchanges were riskier when women were experiencing withdrawal symptoms, as the urge to get money to use drugs prevented them from being selective with clients, and women were more vulnerable to being sexually exploited by clients. Transactional sex in poor conditions and in outdoor venues (e.g., street) also increased women’s vulnerability to sexual risks and violence, as well as being more exposed to social stigmatisation and legal problems [99]. Transactional sex in indoor venues (e.g., hotel) provided women with more opportunities to negotiate condom use, avoid violence and refuse unwanted sexual requests [100]. Transactional sex was also positively associated with having multiple sexual partners—which was linked to a decrease in condom use—, initiating sex at a younger age, and being less likely to have steady sexual partners. These associations, and women’s heightened vulnerability to violence, might explain the poorer sexual health of women engaging in transactional sex [101].

The data around transactional sex and sexual health risks suggest that transactional sex should not be treated as a sexual risk practice but rather a situation in which women are more exposed to sexual risks. These risks are often rooted in the stigmatisation and discrimination of transactional sex workers [102–108] that, together with gender inequities, might be linked to women’s vulnerability to physical and sexual violence by clients. As for any other women, experiencing physical and sexual violence exposes transactional sex workers to sexual health risks. Efforts to prevent STI/BBV transmission among transactional sex workers should then go beyond an individual-level focus and avoid pathologising, victimising and neglecting the needs of this group of women. Structural violence should be considered and addressed in relation to transactional sex. This directly relates to the ongoing debate about the need to revise current outdated legislations that criminalise transactional sex, and contribute to transactional sex workers’ vulnerability to poorer health, exploitative conditions and violence [106, 108–111].

#### HIV Status: What About Stigma and Discrimination?

HIV positive individuals are more likely to use condoms once they are aware of their HIV status [112, 113]. Condomless sex among HIV positive individuals seems to be associated with the increased effectiveness of new treatments for HIV [114]. Sexual transmission of HIV among serodiscordant couples has also been found to be low [115], suggesting high rates of condom use and effectiveness of antiretroviral therapy. Data from this review suggested a tendency for HIV positive women to be more exposed to sexual risks, including sexual violence, compared to HIV negative women. In fact, previous research has drawn attention to the difficulties that HIV positive people experience to use condoms [116–118], and how HIV positive women are susceptible to some high-risk sexual practices after experiencing sexual violence in the context of social conflict [119].

In this review, condom use was found to be encouraged when women’s HIV status was different to their partner’s. Self-reported condom use was also facilitated when women felt safer from STI/BBV transmission by using condoms. Low risk awareness (i.e., believing—or knowing—that partners were STI/HIV negative, and/or believing that they could not transmit or get transmitted HIV) was related to women being less likely to use condoms. In contrast, having had a free HIV antibody test was found to be linked to inconsistent condom use among women engaged in transactional sex. An explanation for this could be that testing might decrease risk awareness, and lead women to have condomless sex. Women with a higher knowledge of HIV/AIDS had less sexual encounters with clients. This suggests that increased knowledge of HIV/AIDS may make women more aware of the sexual health risks they could be exposed to.

Other aspects of HIV transmission, such as the impact of stigma and discrimination, and the fear of diagnosis and disclosure of HIV/AIDS status were not encompassed in the papers included in this review. Stigma and discrimination have been widely studied in relation to HIV/AIDS [120–122] and STIs [123]. They are both barriers for prevention and treatment of HIV [120, 121], and tackling them is crucial for the effectiveness of STI/HIV preventive strategies [123, 124]. Besides, it is important to acknowledge that WHOD experience stigma and discrimination due to the intersectionality of different characteristics of their identity (i.e., female gender, race, sexual orientation, drug use, engagement in transactional sex, homelessness). Hence, stigma and discrimination should not be seen as unidimensional but rather as multidimensional and complex social and structural phenomenon [102–104, 125] that should be addressed in STI/BBV policies and services.

#### Sexual Orientation: Addressing Social and Health Inequities

Women from the Lesbian, Gay, and Bisexual (LGB) community experience social and health inequities [126–133]. These comprise poorer mental health [128–132], substance use [129–131] and physical health including STIs [133]. Health inequities can be explained by the extended heteronormality in the healthcare system (and society), an unequal access to health services, and health professionals’ negative attitudes [126, 127]. Consistent with recent research [126, 134], the findings from this review suggest that LGB women experience higher sexual risks, in comparison with their heterosexual counterparts. These health inequities should be recognised and integrated in STI/BBV preventive strategies, in order to promote social justice and address the specific vulnerabilities and inequities experiences by LGB women.

#### Partner Characteristics, Preferences and Negotiation of Condom Use: Missing the Role of Culture

Several papers presented self-reported barriers and facilitators of condom use in relation to partner’s characteristics such as physical appearance, attractiveness, sexual health history, drug practices/history, and health status. These barriers are consistent with previous research on the barriers to condom use [135–138]. Other barriers and facilitators were linked to preferences, negotiation, skills and availability of condoms. Condom use was facilitated when women discussed their use with their partners, and when there was an agreement on using condoms. Women were more likely to report condom use if they were feeling in control over the decision of having sex with condoms. In turn, using condoms increased the feeling of personal responsibility among women. Other barriers to condom use were being unskilled in negotiating and using condoms, perceiving a decrease of sexual pleasure when using condoms, and women’s or their partner’s dislike of condoms. Some women reported feeling uncomfortable talking about sex, and some partners would directly refuse sex with condoms, a situation that created a challenge for women to negotiate condom use and care for their sexual health. Having a drug-using partner was associated with condomless sex. Condoms were less likely to be used when sex was not planned as condoms were potentially not available in that situation. Moreover, condomless sex was common in situations in which condoms were not available or women could not afford to buy them.

These findings are consistent with previous research [139–142], and they relate to the positive impact of self-efficacy and communication on condom use, as well as the importance of empowering women to negotiate and gain control over sexual health decision-making processes. Also, partner characteristics, preferences and decision-making should be considered as factors associated with condom use. Other individual-level (e.g., personality and cognitive processes) and social correlates (e.g., social norms and cultural perspectives on condoms) are not reflected in these findings and these should be further explored [143]. The impact of culture in health and health behaviours has been especially neglected within health services research and health interventions [144–147]. Culture plays a crucial role in the use of condoms since attitudes and taboos in relation to sex and sexual health, social norms, gendered social roles and power dynamics also shape women’s and their partners’ condom use [148, 149]. Furthermore, it is important to consider that all the included papers in this review exclusively researched on male condoms. Research and strategies for STI/BBV prevention should abandon the supremacy of male condom use over promoting the use of both female and male condoms. This might allow women to counteract the unequal power dynamics between women and men, by increasing women’s control of their sexual health [150–152].

#### Substance Use: Contextual Factors of Drug and Alcohol Use and Sexual Risks

Among women who did not engage in transactional sex, the relationship between condom use and substance use was unclear. The number of sexual partners and scope of sexual acts were however higher when women used drugs. The use of condoms was also related to the sexual partner’s drug using practices. Condomless sex seemed to occur among steady relationships formed by a person who does not use drugs and a PWID, as well as in relationships between a PWNID and a PWID. Substance use, and particularly injecting drug use, was related to engaging in transactional sex. Transactional sex was more common when women experienced withdrawal symptoms, a situation when women were also less selective with their clients. In turn, drugs were often used to cope with transactional sex, and using drugs with clients was associated with a higher vulnerability to violence.

Sexual risks seemed to be associated with the contexts of drug using practices, rather than the use of substances per se. Women often engaged in transactional sex to support their drug use—and sometimes their partners’—and this was more common when in withdrawal. On some occasions women would recur to using drugs to cope with transactional sex events. Data suggest that drug use maintained the engagement in transactional sex, and being involved in transactional sex maintained women’s drug use. This made women more vulnerable to experiencing violence, and significantly increased sexual health risks [60, 106, 153–157]. Providing alternative opportunities (e.g., assist women accessing benefits) and empowering women might enable them to break this pattern. On the other hand, decisions on condom use seemed to differ depending on women’s and their partners’ drug using practices. For this reason, it is key to acknowledge the dynamics between women’s and their partners’ drug using patterns, rather than considering them in isolation. Sexual risks associated with substance use should then be considered from a broader social ecological framework, so that socio-structural factors of substance use are accounted for.

#### Love and Trust: Intimacy and Condom Use

Love and trust were common in steady relationships. These feelings hindered condom use with partners, and facilitated using condoms with clients among women engaging in transactional sex, as condoms were perceived as a barrier for intimacy. In turn, reduced love and trust made women less likely to use condoms with clients [158–162]. Condoms were used for transactional sex as an emotional barrier and coping mechanism. Among transactional sex workers, condomless sex was reserved for romantic relationships to reach intimacy and show love and trust in their partners and in the relationship. These data provide evidence of the importance of multilevel analyses of emotional dynamics in relationships with transactional sex clients and non-clients among WHOD, and the impact of these factors on sexual practices and STI/BBV risk [163].

#### Women and Motherhood: Any Woman’s Preferences and Needs

Following previous research, data from this review suggested that women who wanted to have a baby were likely to engage in condomless sex. Those who wanted to prevent pregnancy used condoms more consistently [164]. Sterilised women were less likely to report condom use [165–167], which may indicatethat women might be more aware and inclined to prevent pregnancy and underestimate the risks of infection. It is important to acknowledge that these findings can be extrapolated to any other women. Even though WHOD might have different needs compared to other groups of women, they should not be pathologised and their needs and rights as *women* should not be neglected. Likewise, it should be recognised that some of the factors identified in this review are not necessarily related to women’s drug using practices but common to any woman.

### Aim 2: The Nature and Quality of the Evidence

#### Study Design and Methodology

It is important to highlight that the direction and role of the factors identified were unclear in most cases. The network of interrelations between factors and outcomes is also imprecise and inconclusive. An explanation for this is that most studies were cross-sectional, and the few longitudinal studies did not focus on exploring the impact of factors on sexual risks over time. For this reason, the findings presented in this review cannot be considered *determinants*, but rather *factors* that are related (or not) to certain sexual risks. The evidence found is highly heterogeneous due to the extensive methodological differences between studies, and the variety in the samples and other study characteristics, making it difficult to synthesise the data. Even though all papers comprised WHOD, the characteristics of the samples were rather diverse. For instance, some studies exclusively included incarcerated women, PWID, or women engaging in transactional sex.

Data were mainly self-reported, which may lead to recall biases and a potential gap between reported and actual behaviour [168, 169]. It also suggests that women might have under-reported sexual risks leading to biased outcomes. It is then clear that merging all data together is not only challenging, but it is important to be cautious and not interpret the findings as from a homogeneous dataset. Future research should include longitudinal and experimental studies, in order to explore the direction of the impact of the identified factors on sexual risks, and compare such findings between different groups (e.g., transactional sex workers vs non-transactional sex workers) and women in different countries and cultures. Also, future studies should carefully approach and address research biases (e.g., self-report bias), and aim at building more homogeneous and comparable evidence.

On the other hand, it remains unknown whether quantitative papers reported all null findings. Criticism of the *p* value and reporting ‘statistically significant results’ only is nothing new [170], and has even led to the ban of ‘null hypothesis significant methods’ in scientific journals [171]. Taking these critiques on board, and following the example of some of the papers included in this review, future research should aim at reporting non-significant results. This could help reach a higher consistency and robustness in the evidence available, as well as to determine which areas need further investigation.

#### Use of Theory and Scope of the Research

The lack of strong theoretical and methodological approaches in the included papers is concerning. Theories are a systematic way of understanding behaviour and different phenomenon, and serve as tools to explain and predict events or situations by specifying relations among factors. They are key to understand the determinants of health and factors associated with sexual risks, as well as to suggest ways to develop effective behaviour change methods [30, 172, 173]. Future research could incorporate theory to bridge the gap between research and practice, aiming to improve the development and implementation of public health interventions for preventing STIs and BBVs.

Social ecological approaches to sexual risks are needed in order to address social and health inequities among WHOD, and develop effective and inclusive STI/BBV preventive strategies [13, 42–45]. This will go beyond individual risk and intra/interpersonal factors and explore the wider determinants of health and socio-structural factors (i.e., the wider social, economic, political and cultural context). Most research included in this review has been conducted in developed western countries, where the social perspectives on sexuality and gender dynamics might differ vastly from those ones in other countries and cultures. Even though exploring cultural [29] and religious [174–176] factors are crucial for STI/BBV prevention, there is no evidence on how culture and religion have an impact on sexual risks among WHOD. Punitive laws, policies and practices violating human rights (e.g., deportation of HIV-positive persons), and the criminalisation of transactional sex and drug use are still a reality in some countries [5, 7, 177]. These have been pinpointed to be powerful barriers to STI/BBV prevention, highlighting the importance to consider country-specific social, economic, environmental and political realities [178]. Therefore, a more comprehensive approach would help us to better understand the interplay of factors that lead to sexual (health) risk among drug-using women.

#### Beyond the Male Condom and HIV

None of the publications included explored the use of female condoms or other barrier methods such as the dental dam, nor the use of pre-exposure prophylaxis (PrEP). All research included in this review exclusively appraised the use of male condoms. Even though research on the female condom and dental dam is limited, research has highlighted the potential benefits of these barrier methods [150, 179–183]. Likewise, advances on the use of pre-exposure prophylaxis (PrEP) seem to be promising in preventing HIV [184–186]. Considering these approaches and methods of prevention will be key for future research and to improve STI/BBV preventive efforts.

Finally, little attention has been paid to STIs and other BBVs in comparison with HIV. Future research should also go beyond HIV infection to provide a wider picture of how STI/BBV-related factors have an impact on WHOD’s sexual health and wellbeing.

## Conclusions and Limitations

This is the first systematic literature review that presents a comprehensive overview of the evidence available on the factors of sexual risks among WHOD, in relation to STI/BBV sexual transmission. Synthesising the data presented several challenges that highlighted the lack of consistency in the methodology and outcomes of the included studies. This review was limited by only including English language papers, self-reporting and reporting biases, and the potential incomplete retrieval of relevant research. The search may have limited the findings as structural factors, partly because policy documents were not purposively searched for. Also, merging qualitative and quantitative data, studies with different samples and methodologies, and the limited use of theory, limited the generalisability of this review.

Overall, this review highlights the interrelation of multiple factors associated with sexual risks and the risk for STIs and BBVs among WHOD. It has also identified crucial implications for future research that might serve as guidance for the development of health promotion strategies to tackle STIs, HIV and other BBVs among WHOD.

## Electronic supplementary material

Below is the link to the electronic supplementary material.

Supplementary material 1 (DOCX 86 kb)

## Footnotes

1 Sexual risks were defined to be women’s engagement in sexual risk practices (e.g., condomless sex), or the exposure to situations in which women were vulnerable to sexual health risks (e.g., sexual violence).

2 The term *transactional sex* is used in this review to define the exchange of sex for money and/or goods, including drugs. As cited by McMillan et al. “(…) the terminology of transactional sex is employed to indicate that particular people who exchange sex for payment would be misrepresented or unnecessarily stigmatized by categorization as prostitutes or sex workers. A need for differentiation from sex work has been asserted on the grounds that the sexual transactions are a financial necessity and a desperate measure, or are opportunistic and temporary responses to limited income options, or that they are non-commercial as the exchange enacts symbolic rather than financial functions. (…)” [79].

3 Not using condoms for all sexual encounters throughout the whole sexual act, or for any sexual activity.

4 Sexual and/or romantic relationship that is maintained over time.

5 Using condoms for all sexual encounters throughout the whole sexual act, or for any sexual activity.

## References

[1] Cavanaugh CE, Hedden SL, Latimer WW (2010). Sexually transmitted infections among pregnant heroin- or cocaine-addicted women in treatment: the significance of psychiatric co-morbidity and sex trade. Int J STD AIDS.

[2] Des Jarlais DC, Arasteh K, McKnight C, Hagan H, Perlman DC, Semaan S (2011). Associations between herpes simplex virus type 2 and HCV with HIV among injecting drug users in New York City: the current importance of sexual transmission of HIV. Am J Public Health.

[3] Edelman NL, Patel H, Glasper A, Bogen-Johnston L (2014). Sexual health risks and health-seeking behaviours among substance-misusing women. J Adv Nurs.

[4] Hwang LY, Zack C, Rickman K, Holleman M (2000). Prevalence of sexually transmitted infections and associated risk factors among populations of drug ubusers. Clin Infect Dis.

[5] UNAIDS (2014). 90-90-90: an ambitious treatment target to help end the AIDS epidemic.

[6] UNAIDS (2016). Do no harm—health, human rights and people who use drugs.

[7] UNAIDS (2015). On the fast-track to end AIDS: 2016-2021 strategy.

[8] World Health Organization. Sexually transmitted infections (STIs). Fact sheet, August 2016: World Health Organization. 2016. http://www.who.int/mediacentre/factsheets/fs110/en/. Accessed July 2017.

[9] World Health Organization (2016). Global health sector strategy on sexually transmitted infections, 2016-2021. Towards ending STIs.

[10] World Health Organization (2016). Global health sector strategy on HIV, 2016-2021. Towards ending AIDS.

[11] World Health Organization (2016). Global health sectors strategy on viral hepatitis, 2016-2021. Towards ending viral hepatitis.

[12] World Health Organization (2016). Combating Hepatitis B and C to reach elimination by 2030. Advocacy brief.

[13] El-Bassel N, Terlikbaeva A, Pinkham S (2010). HIV and women who use drugs: double neglect, double risk. Lancet.

[14] Alves GR, Goulart RA, Lara FI, Lucchese L, Vera I (2016). Inconsistent use of condom in non-injecting illicit drug users. Braz J Infect Dis..

[15] Booth RE, Kwiatkowski CF, Chitwood DD (2000). Sex related HIV risk behaviors: differential risks among injection drug users, crack smokers, and injection drug users who smoke crack. Drug Alcohol Depend.

[16] Branson CE, Clemmey P. Heroin use and sexual risk among adolescents in residential treatment. American Psychological Association 2008 Convention. 2008.

[17] Elifson KW, Klein H, Sterk CE (2006). Predictors of sexual risk-taking among new drug users. J Sex Res.

[18] Hagan H, Perlman DC, Des Jarlais DC (2011). Sexual risk and HIV infection among drug users in New York City: a pilot study. Subst Use Misuse.

[19] Lee TS, Chen YP, Chang CW (2011). Gender differences in the perceived self-efficacy of safer HIV practices among polydrug abusers in Taiwan. Compr Psychiatry.

[20] Strathdee SA, Stockman JK (2010). Epidemiology of HIV among injecting and non-injecting drug users: current trends and implications for interventions. Curr HIV/AIDS Rep.

[21] Friedman SR, Mateu-Gelabert P, Ruggles KV, Goodbody E, Syckes C, Jessell L (2017). Sexual risk and transmission behaviors, partnerships and settings among young adult nonmedical opioid users in New York City. AIDS Behav.

[22] El-Bassel N, Gilbert L, Wu E, Go H, Hill J (2005). HIV and intimate partner violence among methadone-maintained women in New York City. Soc Sci Med.

[23] Kilpatrick DG, Acierno R, Resnick HS, Saunders BE, Best CL (1997). A 2-year longitudinal analysis of the relationships between violent assault and substance use in women. J Consult Clin Psychol.

[24] UNAIDS (2016). HIV prevention among adolescent girls and young women. Putting HIV prevention among adolescent girls and young qomwn on the Fast-Track and engaging men and boys.

[25] Brooks A, Meade CS, Potter JS, Lokhnygina Y, Calsyn DA, Greenfield SF (2010). Gender differences in the rates and correlates of HIV risk behaviors among drug abusers. Subst Use Misuse.

[26] Duff P, Tyndall M, Buxton J, Zhang R, Kerr T, Shannon K (2013). Sex-for-crack exchanges: associations with risky sexual and drug use niches in an urban Canadian city. Harm Reduct J.

[27] Folch C, Fernández-Dávila P, Ferrer L, Soriano R, Díez M, Casabona J (2016). Erratum to “High prevalence of drug consumption and sexual risk behaviors in men who have sex with men”. Medicina Clínica (English Edition).

[28] Mitchell MM, Latimer WW (2009). Unprotected casual sex and perceived risk of contracting HIV among drug users in Baltimore, Maryland: evaluating the influence of non-injection versus injection drug user status. AIDS Care.

[29] Airhuhenbuwa CO, Ford CL, Iwelunmor JI (2014). Why culture matters in health interventions: lessons learned from HIV/AIDS stigma and NCDs. Health Educ Behav.

[30] Glanz K, Bishop DB (2010). The role of behavioral science theory in development and implementation of public health interventions. Annu Rev Public Health.

[31] World Health Organization (2013). The economics of the social determinants of health and health inequalities: a resources book.

[32] Sallis JF, Owen N, Fisher EG (2008). Ecological models of health behavior.

[33] Mayer KH, Pizer HF (2008). HIV prevention: a comprehensive approach.

[34] CDC (1981). Morbidity and mortality weekly report (MMWR).

[35] Kral AH, Bluthenthal RN, Lorvick J, Gee L, Bacchetti P, Edlin BR (2001). Sexual transmission of HIV-1 among injection drug users in San Francisco, USA: risk-factor analysis. Lancet.

[36] Steen R, Elvira Wi T, Kamali A, Ndowa F (2009). Control of sexually transmitted infections and prevention of HIV transmission: mending a fractured paradigm. Bull World Health Organ.

[37] Strathdee SA, Sherman SG (2003). The role of sexual transmission of HIV infection among injection and non-injection drug users. J Urban Health..

[38] Des Jarlais DC, Arasteh K, Perlis TE, Hagan H, Abdul-Quader A, Heackathorn DD (2007). Convergence of HIV seroprevalence among injecting and non-injecting drug users in New York City. Aids.

[39] Aral SO, Over M, Manhart L, Holmes KK, Jamison DT, Breman JG, Measham AR, Alleyne G, Claeson M, Evans DB (2006). Sexually transmitted infections. Disease control priorities in developing countries.

[40] WHO/UNAIDS (2008). Consultation on STI interventions for preventing HIV: appraisal of the evidence.

[41] Trepo C (2014). A brief history of hepatitis milestones. Liver Int.

[42] Baral C, Logie CH, Grosso A, Wirtz AL, Beyrer C (2013). Modified social ecological model: a tool to guide the assessment of the risks and risk contexts of HIV epidemics. BMC Public Health..

[43] McLeroy KR, Bibeau D, Steckler A, Glanz K (1988). An ecological perspective on health promotion programs. Health Educ Behav.

[44] Wellings K, Collumbien M, Slaymaker E, Singh S, Hodges Z, Patel D (2006). Sexual behaviour in context: a global perspective. Lancet.

[45] Degenhardt L, Mathers B, Vickerman P, Rhodes T, Latkin C, Hickman M (2010). Prevention of HIV infection for people who inject drugs: why individual, structural, and combination approaches are needed. Lancet.

[46] Moher D, Liberati A, Tetzlaff J, Altman DG, The PRISMA Group (2009). Preferred reporting items for systematic reviews and meta-analyses: the PRISMA statement. PLoS Med.

[47] Kmet LM, Lee RC, Cook LS (2004). Standard quality assessment criteria for evaluation primary research papers from a variety of fields.

[48] Altman DG (1991). Practical statistics for medical research.

[49] Tyndall MW, Patrick D, Spittal P, Li K, Shaughnessy MV, Schechter MT (2002). Risky sexual behaviours among injection drugs users with high HIV prevalence: implications for STD control. Sex Transm Infect.

[50] Mackesy-Amiti ME, Noodram B, Williams C, Ouellet LJ, Broz D (2013). Sexual risk behavior associated with transition to injection among young non-injecting heroin users. AIDS Behav.

[51] Gu J, Chen H, Chen X, Lau JTF, Wang R, Liu C (2008). Severity of drug dependence, economic pressure and HIV-related risk behaviors among non-institutionalized female injecting drug users who are also sex workers in China. Drug Alcohol Depend.

[52] Gaines TL, Rudolph AE, Brouwer KC, Strathdee SA, Lozada R, Martinez G (2013). The longitudinal association of venue stability with consistent condom use among female sex workers in two Mexico-USA border cities. Int J STD AIDS.

[53] Syvertsen JL, Robertson Bazzi A (2015). Sex work, heroin injection and HIV risk in Tijuana: a love story. Anthropol Conscious.

[54] Carlson RG (1999). ‘Boy’ and ‘girl’: The AIDS risk implications of heroin and cocaine symbolism among injection drug users. Anthropol Med..

[55] Albertin-Carbo P, Domingo-Salvany A, Hartnoll RL (2001). Psychosocial considerations for the prevention of HIV infection in injecting drug users. Qual Health Res.

[56] Epele ME (2002). Gender, violence and HIV: Women’s survival in the streets. Cult Med Psychiatry.

[57] Hansen H, Lopez-Iftikhar MM, Alegria M (2002). The economy of risk and respect: accounts by Puerto Rican sex workers of HIV risk taking. J Sex Res.

[58] Lee TS, Fu LA, Fleming P (2005). Using focus groups to investigate the educational needs of female injecting heroin users in Taiwan in relation to HIV/AIDS prevention. Health Educ Res.

[59] Lam NT (2008). Drugs, sex and AIDS: Sexual relationships among injecting drug users and their sexual partners in Vietnam. Cult Health Sex.

[60] Gossop M, Powis B, Griffiths P, Strang J (1995). Female prostitutes in south London: use of heroin, cocaine and alcohol, and their relationship to health risk behaviors. AIDS Care.

[61] Nyamathi AM, Lewis C, Leake B, Flaskerud J, Bennett C (1995). Barriers to condom use and needle cleaning among impoverished minority female injection drug users and partners of injection drug users. Public Health Rep.

[62] Grella CE, Anglin D, Annon JJ (1996). HIV risk behaviors among women in methadone maintenance treatment. Subst Use Misuse.

[63] El-Bassel N, Gilbert L, Schilling RF, Wada T (2000). Drug abuse and partner violence among women in methadone treatment. J Fam Violence.

[64] Gilbert L, El-Bassel N, Schilling RF, Wada T, Bennet B (2000). Partner violence and sexual HIV risk behaviors among women in methadone treatment. AIDS Behav.

[65] Tortu S, McMahon J, Hamid R, Neaigus A (2000). Drug-using women’s sexual risk: an event analysis. AIDS Behav.

[66] Gore-Felton C, Somlai AM, Benotsch EG, Kelly JA, Ostrovski D, Kozlov A (2003). The influence of gender on factors associated with HIV transmission risk among young Russian injection drug users. Am J Drug Alcohol Abuse.

[67] Wang Q, Lin G (2003). Sex exchange and HIV-related risk behaviors among female heroin users in China. J Drug Issues..

[68] Bell AV, Ompad D, Sherman SG (2006). Sexual and drug risk behaviors among women who have sex with women. Am J Public Health.

[69] Cavanaugh CE, Floyd LJ, Penniman TV, Hulbert A, Gaydos C, Latime WW (2011). Examining racial/ethnic disparities in sexually transmitted diseases among recent heroin-using and cocaine-using women. J Womens Health..

[70] Peng EYC, Yeh CY, Cheng SH, Morisky DE, Lan YC, Chen YMA (2011). A case-control study of HIV infection among incarcerated female drug users: impact of sharing needles and having drug-using sexual partners. J Formos Med Assoc.

[71] Goldenberg SM, Rangel G, Staines H, Vera A, Lozada R, Nguyen L (2013). Individual, interpersonal, and socia-structural correlated of involuntary sex exchange among female sex workers in two Mexico-US border cities. J Acquir Immune Defic Syndr.

[72] Iversen J, Dolan K, Ezard N, Maher L (2015). HIV and Hepatitis C virus infection and risk behaviors among heterosexual, bisexual, and lesbian women who inject drugs in Australia. LGBT Health.

[73] Miller CL, Spittal P, LaLiberte N, Li K, Tyndall M, O’Shaughnessy MV (2002). Females experiencing sexual and drug vulnerabilities are at elevated risk for HIV infection among youth who use injection drugs. J Acquir Immune Defic Syndr.

[74] Miller M, Neaigus A (2002). Sex partner support, drug use and sex risk among HIV-negative non-injecting drug users. AIDS Care.

[75] Medrano MA, Hatch JP, Zule WA, Desmond DP (2003). Childhood trauma and adult prostitution behavior in a multi-ethnic heterosexual drug using population. Am J Drug Alcohol Abuse.

[76] Sanchez J, Comerford M, Chitwood DD, Fernandez MI, McCoy CB (2002). High risk sexual behaviors among heroin sniffers who have no history of injection drug use: implications for HIV risk reduction. AIDS Care.

[77] Syvertsen JL, Robertson Bazzi A, Martinez G, Rangel MG, Ulibarri MD, Fergus KB (2015). Love, trust, and HIV risk among female sex workers and their intimate male partners. Am J Public Health.

[78] Gu J, Wang R, Chen H, Lau JT, Zhang L, Hu X (2009). Prevalence of needle sharing commercial sex behaviors and associated factors in Chinese male and female injecting drug user populations. AIDS Care.

[79] McMillan K, Worth H, Rawstorne P (2018). Usage of the terms prostitution, sex work, transactional sex, and survival sex: their utility in HIV prevention research. Arch Sex Behav..

[80] Campbell AN, Tross S, Dworkin SL, Hu MC, Manuel J, Pavlicova M (2009). Relationship power and sexual risk among women in community-based substance abuse treatment. J Urban Health.

[81] MacRae R, Aalto E (2000). Gendered power dynamics and HIV risk in drug-using sexual relationships. AIDS Care.

[82] Chermack ST, Fuller BE, Blow FC (2000). Predictors of expressed partner and non-partner violence among patients in substance abuse treatment. Drug Alcohol Depend..

[83] Chermack ST, Walton MA, Fuller BE, Blow FC (2001). Correlates of expressed and received violence across relationship types among men and women substance abusers. Psychol Addict Behav.

[84] Chermack ST, Grogan-Kaylor A, Perron BE, Murray RL, De Chavez P, Walton MA (2010). Violence among men and women in substance use disorder treatment: a multi-level event-based analysis. Drug Alcohol Depend.

[85] Stuart GL, Moore TM, Elkins SR, O’Farrell TJ, Temple JR, Ramsey S (2013). The temporal association between substance use and intimate partner violence among women arrested for domestic violence. J Consult Clin Psychol.

[86] Friend F, Langhinrichsen-Rohling J, Eichold BH (2011). Same-day substance use in men and women charged with felony domestic violence offenses. Crim Justice Behav..

[87] Maffli E, Zumbrunn A (2003). Alcohol and domestic violence in a sample of incidents reported to the policy in Zurich City. Subst Use Misuse.

[88] El-Bassel N, Gilbert L, Witte S, Wu E, Chang M (2011). Intimate partner violence and HIV among drug-involved women: contexts linking these two epidemics–challenges and implications for prevention and treatment. Subst Use Misuse.

[89] Girchenko P, King EJ (2017). Correlates of double risk of HIV acquisition and transmission among women who inject drugs in St. Petersburg, Russia. AIDS Behav.

[90] Jewkes RK, Dunkle KL, Nduna M, Shai N (2010). Intimate partner violence, relationship power inequity, and incidence of HIV infection in young women in South Africa: a cohort study. Lancet..

[91] Peasant C, Sullivan TP, Weiss NH, Martinez I, Meyer JP (2017). Beyond the syndemic: condom negotiation and use among women experiencing partner violence. AIDS Care.

[92] Montesanti SR (2015). The role of structural and interpersonal violence in the lives of women: a conceptual shift in prevention of gender-based violence. BMC Women’s Health.

[93] Michau L, Horn J, Bank A, Dutt M, Zimmerman C (2015). Prevention of violence against women and girls: lessons from practice. Lancet..

[94] Heise L (1998). Violence against women: an integrated, ecological framework. Violence Against Women.

[95] Wingood GM, DiClemente RJ (2000). Application of the theory of gender and power to examine HIV-related exposures, risk factors, and effective interventions for women. Health Educ Behav..

[96] Connell RW (1987). Gender and power.

[97] Heise L (2011). What works to prevent partner violence: an evidence overview.

[98] Yuen WW, Tran L, Wong CK, Holroyd E, Tang CS, Wong WC (2016). Psychological health and HIV transmission among female sex workers: a systematic review and meta-analysis. AIDS Care..

[99] Sanders T (2016). The risks of street prostitution: punters. Police and protesters. Urban Stud.

[100] Krusi A, Chettiar J, Ridgway A, Abbott J, Strathdee SA, Shannon K (2012). Negotiating safety and sexual risk reduction with clients in unsactioned safer indoor sex work environments: a qualitative study. Am J Public Health.

[101] Mc Grath-Lone L, Marsh K, Hughes G, Ward H (2014). The sexual health of female sex workers compared with other women in England: analysis of cross-sectional data from genitourinary medicine clinics. Sex Transm Infect.

[102] Logie CH, James L, Tharao W, Loutfy MR (2011). HIV, gender, race, sexual orientation, and sex work: a qualitative study of intersectional stigma experienced by HIV-positive women in Ontario, Canada. PLoS Med.

[103] Lacombe-Duncan A (2016). An intersectional perspective on access to HIV-related healthcare for transgender women. Transgend Health.

[104] Wagner AC, Girard T, McShane KE, Margolese SL, Hart TA (2017). HIV-related stigma and overlapping stigmas towards people living with HIV among health care trainees in Canada. AIDS Educ Prev.

[105] King EJ, Maman S, Bowling JM, Moracco KE, Dudina V (2013). The influence of stigma and discrimination on female sex workers’ access to HIV services in St. Petersburg, Russia. AIDS Behav.

[106] Weitzer R (2009). Sociology of sex work. Ann Rev Sociol.

[107] Scambler G (2016). Sex work stigma: opportunist migrants in London. Sociology.

[108] Decker MR, Crago A-L, Chu SKH, Sherman SG, Seshu MS, Buthelezi K (2015). Human rights violations against sex workers: burden and effect on HIV. Lancet..

[109] Morton A, Tabrizi S, Garland S, Lee P, Reid P, Fairley C (2002). Will the legalisation of street sex work improve health?. Sex Transm Infect..

[110] Hubbard P, Matthews R, Scoular J (2008). Regulating sex work in the EU: prostitute women and the new spaces of exclusion. Gend, Place Cult.

[111] Baratosy R, Wendt S (2017). “Outdated Laws, Outspoken Whores”: exploring sex work in a criminalised setting. Women’s Stud Int Forum.

[112] Holtgrave DR, Hall HI, Wehrmeyer L, Maulsby C (2012). Costs, consequences and feasibility of strategies for achieving the goals of the National HIV/AIDS strategy in the United States: a closing window for success?. AIDS Behav.

[113] Allen S, Meinzen-Derr J, Kautzman M, Zulu I, Trask S, Fideli U (2003). Sexual behavior of HIV discordant couples after HIV counseling and testing. Aids..

[114] Van der Straten A, Gómez CA, Saul J, Quan J, Padian N (2000). Sexual risk behaviors among heterosexual HIV serodiscordant couples in the era of post-exposure prevention and viral suppressive therapy. Aids..

[115] Attia S, Egger M, Müller M, Zwahlen M, Low N (2009). Sexual transmission of HIV according to viral load and antiretroviral therapy: systematic review and meta-analysis. Aids..

[116] Schiltz MAST (2000). HIV-positive people, risk and sexual behavior. Soc Sci Med.

[117] Kalichman SC (1999). Psychological and social correlates of high-risk sexual behaviour among men and women living with HIV/AIDS. AIDS Care.

[118] Kalichman SC, Rompa R, Cage M, Di Fonzo K, Simpson D, Austin J (2001). Effectiveness of an intervention to reduce HIV trasmission risks in HIV-positive people. Am J Prev Med.

[119] Adedimeji AA, Hoover DR, Shi Q, Gard T, Mutimura E, Sinayobye J (2015). Sexual behavior and risk practices of hiv positive and HIV negative Rwandan women. AIDS Behav.

[120] Mahajan AP, Sayles JN, Patel VA, Remien RH, Ortiz D, Szekeres G (2008). Stigma in the HIV/AIDS epidemic: a review of the literature and recommendations for the way forward. AIDS (London, England).

[121] Stangl AL, Lloyd JK, Brady LM, Holland CE, Baral S (2013). A systematic review of interventions to reduce HIV-related stigma and discrimination from 2002 to 2013: how far have we come?. J Int AIDS Soc..

[122] Chambers LA, Rueda S, Baker DN, Wilson MG, Deutsch R, Raeifar E (2015). Stigma, HIV and health: a qualitative synthesis. BMC Public Health..

[123] Hood JE, Friedman AL (2011). Unveiling the hidden epidemic: a review of stigma associated with sexually transmissible infections. Sex Health..

[124] Piot P (2006). AIDS: from crisis management to sustained strategic response. Lancet.

[125] Earnshaw VA, Smith LR, Cunningham CO, Copenhaver MM (2015). Intersectionality of internalized HIV stigma and internalized substance use stigma: implications for depressive symptoms. J Health Psychol..

[126] Logie CH, Lacombe-Duncan A, Wang Y, Kaida A, de Pokomandy A, Webster K (2017). Sexual orientation differences in health and wellbeing among women living with HIV in Canada: findings from a National Cohort Study. AIDS Behav..

[127] Fish J, Bewley S (2010). Using human rights-based approaches to conceptualise lesbian and bisexual women’s health inequalities. Health Soc Care Community.

[128] King M, Semlyen J, See Tai S, Killaspy H, Osborn D, Popelyuk D (2008). A systematic review of mental disorder, suicide, and deliberate self-harm in lesbian, gay and bisexual people. BMC Psychiatry..

[129] Operario D, Gamarel KE, Grin BM, Lee JH, Kahler CW, Marsall BDL (2015). Sexual minority health disparities in adult men and women in the United States: National Health and Nutrition Examination Survey, 2001-2010. Am J Public Health..

[130] Hughes TL, Johnson TP, Steffen AD, Wilsnack SC, Everett B (2014). Lifetime victimization, hazardous drinking, and depression among heterosexual and sexual minority women. LGBT Health..

[131] Button DM, O’Connell DJ, Gealt R (2012). Sexual minority youth victimization and social support: the intersection of sexuality, gender, race and victimization. J Homosex.

[132] Pyra M, Weber KM, Wilson TE, Cohen J, Murchison L, Goparaju L (2014). Sexual minority women and depressive symptoms throughout adulthood. Am J Public Health..

[133] Fethers K, Marks C, Mindel A, Estcourt CS (2000). Sexually transmitted infections and risk behaviours in women who have sex with women. Sex Transm Infect.

[134] Lyons T, Kerr T, Duff P, Feng C, Shannon K (2014). Youth, violence and non-injecting drug use: nexus of vulnerabilities among lesbian and bisexual sex workers. AIDS Care.

[135] Nehl EJ, Elifson K, DePadilla L, Sterk C (2016). Sex partner type, drug use and condom use self-efficacy among African Americans from disadvantaged neighborhoods: are associations with consistent condom use moderated by gender?. J Sex Res.

[136] Noar SM, Carlyle K, Cole C (2006). Why communication is crucial: Meta-analysis of the relationship between safer sexual communication and condom use. J Health Commun..

[137] Rinehart JK, Yeater EA (2012). The effects of male attractiveness and sexual attitudes on women’s risk perception. Violence Against Women.

[138] Sterk CE, Klein H, Elifson KW (2003). Perceived condom use self-efficacy among at-risk women. AIDS Behav.

[139] Bandura A (1977). Self-efficacy: toward a unifying theory of behavioural change. Psychol Rev.

[140] Campbell AN, Brooks AJ, Pavlicova M, Hu M-C, Hatch-Maillette MA, Calsyn DA (2016). Barriers to condom use: results for men and women enrolled in HIV risk reduction trials in outpatient drug treatment. J HIV/AIDS Soc Serv.

[141] Kapadia F, Latka MH, Wu Y, Strathdee SA, Mackesy-Amiti ME, Hudson SM (2011). Longitudinal determinants of consistent condom use by partner type among young injection drug users: the role of personal and partner characteristics. AIDS Behav.

[142] Sarkar NN (2008). Barriers to condom use. EUR J Contracept Reprod Health Care.

[143] Sheeran P, Abraham C, Orbell S (1999). Psychosocial correlates of heterosexual condom use: a meta-analysis. Psychol Bull.

[144] Asad AL, Kay T (2015). Toward a multidimensional understanding of culture for health interventions. Soc Sci Med.

[145] Singer MK (2012). Applying the concept of culture to reduce health disparities through health behaviour research. Prev Med.

[146] Singer MK, Dressler W, George S, The NIH (2016). Expert panel. Culture: the missing link in health research. Soc Sci Med.

[147] Napier AD, Ancarno C, Butler B, Calabrese J, Chater A, Chatterjee H (2014). Culture and health. Lancet..

[148] Kocken P, van Dorst A, Schaalma H (2006). The relevance of cultural factors in predicting condom-use intentions among immigrants from the Netherlands Antilles. Health Educ Res.

[149] Gómez CA, Marín BV (1996). Gender, culture and power: barriers to HIV-prevention strategies for women. J Sex Res.

[150] Hoffman S, Mantell J, Exner T, Stein Z (2004). The future of the female condom. Perspect Sex Reprod Health..

[151] Stockman JK, Morris MD, Martinez G, Lozada R, Patterson TL, Ulibarri MD (2012). Prevalence and correlates of female condom use and interest among injection drug-using female sex workers in two Mexico-US border cities. AIDS Behav.

[152] Gollub EL (2000). The female condom: tool for women’s empowerment. Am J Public Health.

[153] Strathdee SA, Lozada R, Martinez G, Vera A, Rusch ML (2011). Social and structural factors associates with HIV infection among female sex workers who inject drugs in Mexico-US border region. PLoS ONE.

[154] De Graff R, Vanwesenbeeck I, van Zessen G, Straver CJ, Visser JH (1995). Alcohol and drug use in heterosexual and homosexual prostitution, and its relation to protection behaviour. AIDS Care..

[155] Strathdee SA, Abramovitz D, Lozada R, Martinez G, Gudelia Rangel M, Vera A (2013). Reductions in HIV/STI incidence and sharing of injection equipment among female sex workers who inject drugs: results from randomized controlled trial. PLoS ONE.

[156] Strathdee SA, Philbin MM, Semple SJ, Pu M, Orozovich P (2008). Correlates of injection drug use among female sex workers in two Mexico-US border cities. Drug Alcohol Depend.

[157] Wechsberg WM, Krupitsky E, Romanova T, Zvartau E, Kline TL (2012). Double jeopardy-drug and sex risks among Russian women who inject drugs: initial feasibility and efficacy results of a small randomized controlled trial. Subst Abuse Treat Prev Policy..

[158] Stoebenau K, Hindin MJ, Nathanson CA, Rakotoarison PG, Razafintsalama V (2009). “… But then he became my sipa”: the implications of relationship fluidity for condom use among women sex workers in Antananarivo, Madagascar. Am J Public Health.

[159] Amaro H (1995). Love, sex, and power: considering women’s realities in HIV prevention. Am Psychol.

[160] Lazar C, SanClemente C, Ferrer L, Folch C, Casabona J (2015). Condom use among female sex workers in Catalonia: why do they use a condom, why don’t they use it?. AIDS Educ Prev.

[161] Robertson AM, Syvertsen JL, Amaro H (2014). Can’t buy my love: a typology of female sex workers’ commercial relationships in the Mexico-US border region. J Sex Res.

[162] Sherman SG, Latkin CA (2001). Intimate relationship characteristics associated with condom use among drug users and their sex partners: a multilevel analysis. Drug Alcohol Depend.

[163] Damasio AR (1994). Descartes’ error: emotion, reason, and the human brain.

[164] Cabral RJ, Calavotti C, Armstrong K, Morrow BMS, Fogarty L (2008). Reproductive and contraceptive attitudes as predictors of condom use among women in a HIV prevention intervention. Women Health.

[165] Pruitt SL, von Sternberg K, Velasquez MM, Dolan Mullen P (2010). Condom use among sterilized and nonsterilized women in county jail and residential treatment centers. Women Health Issues.

[166] Sangi-Haghpeykar H, Horth F, Poindexter AN (2001). Condom use among sterilized and nonsterilized Hispanic women. Sex Transm Dis.

[167] Semaan S, Lauby J, Walls C (1997). Condom use with main partners by sterilized and non-sterilized women. Women Health.

[168] Gallo MF, Behets FM, Steiner MJ (2007). Validity of self-reported ‘safe sex’ among female sex workers in Mombasa, Kenya. PSA Analysis. Int J STD AIDS.

[169] Schroder KEE, Carey MP, Vanable PA (2003). Methodological challenges in research on sexual risk behaviour: II, Accuracy of self-reports. Ann Behav Med.

[170] Leek JT, Peng RD (2015). Statistics: p values are just the tip of the iceberg. Nature.

[171] Trafimow D, Marks M (2015). Editorial: basic and applied science. Appl Soc Psychol..

[172] Abraham C, Michie S, Abraham C (2004). Using theory in research. Health psychology in practice.

[173] Marks DF (2008). The quest for meaningful theory in health psychology. J Health Psychol.

[174] Weiss ML, Chitwood DD, Sanchez J (2008). Religiosity, drug use, and HIV-related risk behaviors among heroin injectors. J Drug Issues.

[175] Billioux VG, Sherman SG, Latkin CA (2014). Religiosity and HIV-related drug risk behaviour: a multidimensional assessment of individuals from communities with high rates of drug use. J Relig Health.

[176] Shaw SA, El-Bassel N (2014). The influence of religion on sexual HIV risk. AIDS Behav.

[177] Kalichman SC (2017). Pence, Putin, Mbeki and their HIV/AIDS-related crimes against humanity: call for social justice and behavioural science advocacy. AIDS Behav.

[178] Sumartojo E (2000). Structural factors in HIV prevention: concepts, examples, and implications for research. Aids..

[179] Maksut JL, Eaton LA (2015). Female condoms = missed opportunities: lessons learned from promotion-centered interventions. Women’s Health Issues.

[180] Marfatia YS, Pendya I, Mehta K (2015). Condoms: past, present and future. Indian J Sex Transm Dis..

[181] Ritchers J, Clayton S (2010). The practical and symbolic purpose of dental dams in lesbian safer sex promotion. Sex Health..

[182] Sakondhavat C (2002). Challenges to female condom integration into condom programming. Int J STD AIDS.

[183] Vijayakumar G, Mabude Z, Smit J, Beksinska M, Lurie M (2006). A review of female-condom effectiveness: patterns of use and impact on protected sex acts and STI incidence. Int J STD AIDS.

[184] Baeten JM, Donnell D, Ndase P, Mugo NR, Campbell JD, Wangisi J (2012). Antiretroviral prophylaxis for HIV prevention in heterosexual men and women. N Engl J Med..

[185] Choopanya K, Martin M, Suntharasamai P, Sangkum U, Mock PA, Leethochawalit M (2013). Antiretroviral prophylaxis for HIV infection in injecting drug users in Bangkok, Thailand (the Bangkok Tenofovir Study): a randomised, double-blind, placebo-controlled phase 3 trial. Lancet..

[186] McCormack S, Dunn DT, Desai M, Dolling DI, Gafos M, Gilson R (2016). Pre-exposure prophylaxis to prevent the acquisition of HIV-1 infection (PROUD): effectiveness results from the pilot phase of a pragmatic open-label randomised trial. Lancet..

