# Supplementary material for: Factors Associated with Sexual Risks and Risk of STIs, HIV and Other Blood-Borne Viruses Among Women Using Heroin and Other Drugs: A Systematic Literature Review
Source: AIDS Behav. 2018 Aug 2;23(1):222–51. doi: 10.1007/s10461-018-2238-7 (PMC6342849; doi:10.1007/s10461-018-2238-7)
Supplement: Supplementary file 1 — Supplementary material 1 (DOCX 86 kb) [file 10461_2018_2238_MOESM1_ESM.docx]

|  | **Criteria** | **Yes**  **(2)** | **Partial**  **(1)** | **No**  **(0)** |
| --- | --- | --- | --- | --- |
| 1 | Question / objective sufficiently described? |  |  | x |
| 2 | Study design evident and appropriate? | x |  |  |
| 3 | Context for the study clear? | x |  |  |
| 4 | Connection to a theoretical framework / wider body of knowledge? |  | x |  |
| 5 | Sampling strategy described, relevant and justified? |  | x |  |
| 6 | Data collection methods clearly described and systematic? | x |  |  |
| 7 | Data analysis clearly described and systematic? |  | x |  |
| 8 | Use of verification procedure(s) to establish credibility? |  |  | x |
| 9 | Conclusions supported by the results? | x |  |  |
| 10 | Reflexivity of the account? |  |  | x |

**Supplement A. Quality of included studies**

**Qualitative Studies**

**[50]** Carlson, R. G. (1999). ‘Boy’ and ‘girl’: The AIDS risk implications of heroin and cocaine symbolism among injection drug users. *Anthropology & Medicine, 6* (1), 59-77.

**Total score: 11/20**

**[55]** Albertin-Carbo, P., Domingo-Salvany, A. & Hartnoll, R. L. (2001). Psychosocial considerations for the prevention of HIV infection in injecting drug users. *Qualitative Health Research, 11* (1), 26-39.

|  | **Criteria** | **Yes**  **(2)** | **Partial**  **(1)** | **No**  **(0)** |
| --- | --- | --- | --- | --- |
| 1 | Question / objective sufficiently described? | x |  |  |
| 2 | Study design evident and appropriate? | x |  |  |
| 3 | Context for the study clear? | x |  |  |
| 4 | Connection to a theoretical framework / wider body of knowledge? | x |  |  |
| 5 | Sampling strategy described, relevant and justified? |  | x |  |
| 6 | Data collection methods clearly described and systematic? | x |  |  |
| 7 | Data analysis clearly described and systematic? |  |  | x |
| 8 | Use of verification procedure(s) to establish credibility? |  |  | x |
| 9 | Conclusions supported by the results? | x |  |  |
| 10 | Reflexivity of the account? | x |  |  |

**Total score: 15/20**

**[52]** Epele, M. E. (2002). Gender, violence and HIV: Women’s survival in the streets. *Culture, Medicine and Psychiatry, 26*, 33-54.

|  | **Criteria** | **Yes**  **(2)** | **Partial**  **(1)** | **No**  **(0)** |
| --- | --- | --- | --- | --- |
| 1 | Question / objective sufficiently described? |  | x |  |
| 2 | Study design evident and appropriate? |  | x |  |
| 3 | Context for the study clear? | x |  |  |
| 4 | Connection to a theoretical framework / wider body of knowledge? | x |  |  |
| 5 | Sampling strategy described, relevant and justified? |  | x |  |
| 6 | Data collection methods clearly described and systematic? | x |  |  |
| 7 | Data analysis clearly described and systematic? |  |  | x |
| 8 | Use of verification procedure(s) to establish credibility? |  |  | x |
| 9 | Conclusions supported by the results? | x |  |  |
| 10 | Reflexivity of the account? | x |  |  |

**Total score: 13/20**

**[57]** Hansen, H., Lopez-Iftikhar, M. M. & Alegria, M. (2002). The economy of risk and respect: Accounts by Puerto Rican sex workers of HIV risk taking. *The Journal of Sex Research, 39* (4), 292-301.

|  | **Criteria** | **Yes**  **(2)** | **Partial**  **(1)** | **No**  **(0)** |
| --- | --- | --- | --- | --- |
| 1 | Question / objective sufficiently described? |  | x |  |
| 2 | Study design evident and appropriate? |  | x |  |
| 3 | Context for the study clear? | x |  |  |
| 4 | Connection to a theoretical framework / wider body of knowledge? | x |  |  |
| 5 | Sampling strategy described, relevant and justified? | x |  |  |
| 6 | Data collection methods clearly described and systematic? | x |  |  |
| 7 | Data analysis clearly described and systematic? |  | x |  |
| 8 | Use of verification procedure(s) to establish credibility? | x |  |  |
| 9 | Conclusions supported by the results? | x |  |  |
| 10 | Reflexivity of the account? | x |  |  |

**Total score: 17/20**

|  | **Criteria** | **Yes**  **(2)** | **Partial**  **(1)** | **No**  **(0)** |
| --- | --- | --- | --- | --- |
| 1 | Question / objective sufficiently described? | x |  |  |
| 2 | Study design evident and appropriate? | x |  |  |
| 3 | Context for the study clear? | x |  |  |
| 4 | Connection to a theoretical framework / wider body of knowledge? | x |  |  |
| 5 | Sampling strategy described, relevant and justified? | x |  |  |
| 6 | Data collection methods clearly described and systematic? | x |  |  |
| 7 | Data analysis clearly described and systematic? |  | x |  |
| 8 | Use of verification procedure(s) to establish credibility? |  | x |  |
| 9 | Conclusions supported by the results? | x |  |  |
| 10 | Reflexivity of the account? | x |  |  |

**[58]** Lee, T. S-H., Fu, L-A. & Fleming, P. (2005). Using focus groups to investigate the educational needs of female injecting heroin users in Taiwan in relation to HIV/AIDS prevention. *Health Education Research, 21* (1), 55-65.

**Total score: 18/20**

|  | **Criteria** | **Yes**  **(2)** | **Partial**  **(1)** | **No**  **(0)** |
| --- | --- | --- | --- | --- |
| 1 | Question / objective sufficiently described? | x |  |  |
| 2 | Study design evident and appropriate? | x |  |  |
| 3 | Context for the study clear? | x |  |  |
| 4 | Connection to a theoretical framework / wider body of knowledge? | x |  |  |
| 5 | Sampling strategy described, relevant and justified? |  |  | x |
| 6 | Data collection methods clearly described and systematic? | x |  |  |
| 7 | Data analysis clearly described and systematic? |  | x |  |
| 8 | Use of verification procedure(s) to establish credibility? |  |  | x |
| 9 | Conclusions supported by the results? | x |  |  |
| 10 | Reflexivity of the account? | x |  |  |

**[59]** Lam, N. T. (2008). Drugs, sex and AIDS: Sexual relationships among injecting drug users and their sexual partners in Vietnam. *Culture, Health & Sexuality, 10* (S1), S123-S137.

**Total score: 15/20**

**[53]** Syvertsen, J. L. & Robertson Bazzi, A. (2015). Sex work, heroin injection, and HIV risk in Tijuana: A love story. *Anthropology of Consciousness, 26* (2), 182-194.

|  | **Criteria** | **Yes**  **(2)** | **Partial**  **(1)** | **No**  **(0)** |
| --- | --- | --- | --- | --- |
| 1 | Question / objective sufficiently described? | x |  |  |
| 2 | Study design evident and appropriate? | x |  |  |
| 3 | Context for the study clear? | x |  |  |
| 4 | Connection to a theoretical framework / wider body of knowledge? | x |  |  |
| 5 | Sampling strategy described, relevant and justified? |  |  | x |
| 6 | Data collection methods clearly described and systematic? |  | x |  |
| 7 | Data analysis clearly described and systematic? |  |  | x |
| 8 | Use of verification procedure(s) to establish credibility? |  | x |  |
| 9 | Conclusions supported by the results? | x |  |  |
| 10 | Reflexivity of the account? | x |  |  |

**Total score: 14/20**

**Quantitative Studies**

**[60]** Gossop, M., Powis, B. Griffiths, P. & Strang, J. (1995). Female prostitutes in south London: use of heroin, cocaine and alcohol, and their relationship to health risk behaviours. *AIDS Care, 7* (3), 253-260.

|  | **Criteria** | **Yes**  **(2)** | **Partial**  **(1)** | **No**  **(0)** | **N/A** |
| --- | --- | --- | --- | --- | --- |
| 1 | Question / objective sufficiently described? |  | x |  |  |
| 2 | Study design evident and appropriate? |  | x |  |  |
| 3 | Method of subject/comparison group selection or source of information/input variables described and appropriate? | x |  |  |  |
| 4 | Subject (and comparison group, if applicable) characteristics sufficiently described? |  | x |  |  |
| 5 | If interventional and random allocation was possible, was it described? |  |  |  | x |
| 6 | If interventional and blinding of investigators was possible, was it reported? |  |  |  | x |
| 7 | If interventional and blinding of subjects was possible, was it reported? |  |  |  | x |
| 8 | Outcome and (if applicable) exposure measure(s) well defined and robust to measurement / misclassification bias? Means of assessment reported? | x |  |  |  |
| 9 | Sample size appropriate? | x |  |  |  |
| 10 | Analytic methods described/justified and appropriate? |  |  | x |  |
| 11 | Some estimate of variance is reported for the main results? |  |  | x |  |
| 12 | Controlled for confounding? |  |  |  | x |
| 13 | Results reported in sufficient detail? |  | x |  |  |
| 14 | Conclusions supported by the results? | x |  |  |  |

**Total score: 12 - (4 x 2) = 4/16**

**[61]** Nyamathi, A. M., Lewis, C., Leake, B., Flaskerud, J. & Bennett, C. (1995). Barriers to condom use and needle cleaning among impoverished minority female injection drug users and partners of injection drug users. *Public Health Reports, 110* (2), 166-172.

|  | **Criteria** | **Yes**  **(2)** | **Partial**  **(1)** | **No**  **(0)** | **N/A** |
| --- | --- | --- | --- | --- | --- |
| 1 | Question / objective sufficiently described? | x |  |  |  |
| 2 | Study design evident and appropriate? |  |  | x |  |
| 3 | Method of subject/comparison group selection or source of information/input variables described and appropriate? | x |  |  |  |
| 4 | Subject (and comparison group, if applicable) characteristics sufficiently described? |  | x |  |  |
| 5 | If interventional and random allocation was possible, was it described? |  |  |  | x |
| 6 | If interventional and blinding of investigators was possible, was it reported? |  |  |  | x |
| 7 | If interventional and blinding of subjects was possible, was it reported? |  |  |  | x |
| 8 | Outcome and (if applicable) exposure measure(s) well defined and robust to measurement / misclassification bias? Means of assessment reported? |  | x |  |  |
| 9 | Sample size appropriate? | x |  |  |  |
| 10 | Analytic methods described/justified and appropriate? | x |  |  |  |
| 11 | Some estimate of variance is reported for the main results? |  |  | x |  |
| 12 | Controlled for confounding? |  |  |  | x |
| 13 | Results reported in sufficient detail? |  | x |  |  |
| 14 | Conclusions supported by the results? | x |  |  |  |

**Total score: 13 – (4 x 2) = 5/16**

**[62]** Grella, C. E., Anglin, D. & Annon, J. J. (1996). HIV risk behaviors among women in methadone maintenance treatment. *Substance Use & Misuse, 31* (3), 277-301.

|  | **Criteria** | **Yes**  **(2)** | **Partial**  **(1)** | **No**  **(0)** | **N/A** |
| --- | --- | --- | --- | --- | --- |
| 1 | Question / objective sufficiently described? | x |  |  |  |
| 2 | Study design evident and appropriate? | x |  |  |  |
| 3 | Method of subject/comparison group selection or source of information/input variables described and appropriate? | x |  |  |  |
| 4 | Subject (and comparison group, if applicable) characteristics sufficiently described? | x |  |  |  |
| 5 | If interventional and random allocation was possible, was it described? |  |  |  | x |
| 6 | If interventional and blinding of investigators was possible, was it reported? |  |  |  | x |
| 7 | If interventional and blinding of subjects was possible, was it reported? |  |  |  | x |
| 8 | Outcome and (if applicable) exposure measure(s) well defined and robust to measurement / misclassification bias? Means of assessment reported? | x |  |  |  |
| 9 | Sample size appropriate? | x |  |  |  |
| 10 | Analytic methods described/justified and appropriate? | x |  |  |  |
| 11 | Some estimate of variance is reported for the main results? | x |  |  |  |
| 12 | Controlled for confounding? |  |  |  | x |
| 13 | Results reported in sufficient detail? | x |  |  |  |
| 14 | Conclusions supported by the results? | x |  |  |  |

**Total score: 20 – (4 x 2) = 16/16**

**[63]** El-Bassel, N., Gilbert, L., Schilling, R. & Wada, T. (2000). Drug abuse and partner violence among women in methadone treatment. *Journal of Family Violence, 15* (3), 209-228.

|  | **Criteria** | **Yes**  **(2)** | **Partial**  **(1)** | **No**  **(0)** | **N/A** |
| --- | --- | --- | --- | --- | --- |
| 1 | Question / objective sufficiently described? | x |  |  |  |
| 2 | Study design evident and appropriate? | x |  |  |  |
| 3 | Method of subject/comparison group selection or source of information/input variables described and appropriate? | x |  |  |  |
| 4 | Subject (and comparison group, if applicable) characteristics sufficiently described? | x |  |  |  |
| 5 | If interventional and random allocation was possible, was it described? |  |  |  | x |
| 6 | If interventional and blinding of investigators was possible, was it reported? |  |  |  | x |
| 7 | If interventional and blinding of subjects was possible, was it reported? |  |  |  | x |
| 8 | Outcome and (if applicable) exposure measure(s) well defined and robust to measurement / misclassification bias? Means of assessment reported? | x |  |  |  |
| 9 | Sample size appropriate? | x |  |  |  |
| 10 | Analytic methods described/justified and appropriate? | x |  |  |  |
| 11 | Some estimate of variance is reported for the main results? | x |  |  |  |
| 12 | Controlled for confounding? |  |  |  | x |
| 13 | Results reported in sufficient detail? | x |  |  |  |
| 14 | Conclusions supported by the results? | x |  |  |  |

**Total score: 20 – (4 x 2) = 16/16**

**[64]** Gilbert, L., El-Bassel, N., Schilling, R. F., Wada, T. & Bennet, B. (2000). Partner violence and sexual HIV risk behaviors among women in methadone treatment. *AIDS and Behavior, 4* (3), 261-269.

|  | **Criteria** | **Yes**  **(2)** | **Partial**  **(1)** | **No**  **(0)** | **N/A** |
| --- | --- | --- | --- | --- | --- |
| 1 | Question / objective sufficiently described? | x |  |  |  |
| 2 | Study design evident and appropriate? | x |  |  |  |
| 3 | Method of subject/comparison group selection or source of information/input variables described and appropriate? | x |  |  |  |
| 4 | Subject (and comparison group, if applicable) characteristics sufficiently described? |  |  |  |  |
| 5 | If interventional and random allocation was possible, was it described? |  |  |  | x |
| 6 | If interventional and blinding of investigators was possible, was it reported? |  |  |  | x |
| 7 | If interventional and blinding of subjects was possible, was it reported? |  |  |  | x |
| 8 | Outcome and (if applicable) exposure measure(s) well defined and robust to measurement / misclassification bias? Means of assessment reported? | x |  |  |  |
| 9 | Sample size appropriate? | x |  |  |  |
| 10 | Analytic methods described/justified and appropriate? | x |  |  |  |
| 11 | Some estimate of variance is reported for the main results? | x |  |  |  |
| 12 | Controlled for confounding? |  |  |  | x |
| 13 | Results reported in sufficient detail? | x |  |  |  |
| 14 | Conclusions supported by the results? | x |  |  |  |

**Total score: 20 – (4 x 2) = 16/16**

**[65]** Tortu, S., McMahon, J., Hamid, R. & Neaigus, A. (2000). Drug-using women’s sexual risk: An event analysis. *AIDS and Behavior, 4* (4), 329-340.

|  | **Criteria** | **Yes**  **(2)** | **Partial**  **(1)** | **No**  **(0)** | **N/A** |
| --- | --- | --- | --- | --- | --- |
| 1 | Question / objective sufficiently described? | x |  |  |  |
| 2 | Study design evident and appropriate? |  |  |  |  |
| 3 | Method of subject/comparison group selection or source of information/input variables described and appropriate? | x |  |  |  |
| 4 | Subject (and comparison group, if applicable) characteristics sufficiently described? |  | x |  |  |
| 5 | If interventional and random allocation was possible, was it described? |  |  |  | x |
| 6 | If interventional and blinding of investigators was possible, was it reported? |  |  |  | x |
| 7 | If interventional and blinding of subjects was possible, was it reported? |  |  |  | x |
| 8 | Outcome and (if applicable) exposure measure(s) well defined and robust to measurement / misclassification bias? Means of assessment reported? | x |  |  |  |
| 9 | Sample size appropriate? | x |  |  |  |
| 10 | Analytic methods described/justified and appropriate? | x |  |  |  |
| 11 | Some estimate of variance is reported for the main results? | x |  |  |  |
| 12 | Controlled for confounding? |  |  |  | x |
| 13 | Results reported in sufficient detail? | x |  |  |  |
| 14 | Conclusions supported by the results? | x |  |  |  |

**Total score: 19 – (4 x 2) = 15/16**

**[73]** Miller, C. L., Spittal, P. M., LaLiberte, N., Li, K., Tyndall, M. W., O’Shaughnessy, M. V. & Schechter, M. T. (2002). Females experiencing sexual and drug vulnerabilities are at elevated risk for HIV infection among youth who use injection drugs. *Journal of Acquired Immune Deficiency Syndromes, 30*, 335-341.

|  | **Criteria** | **Yes**  **(2)** | **Partial**  **(1)** | **No**  **(0)** | **N/A** |
| --- | --- | --- | --- | --- | --- |
| 1 | Question / objective sufficiently described? | x |  |  |  |
| 2 | Study design evident and appropriate? | x |  |  |  |
| 3 | Method of subject/comparison group selection or source of information/input variables described and appropriate? | x |  |  |  |
| 4 | Subject (and comparison group, if applicable) characteristics sufficiently described? | x |  |  |  |
| 5 | If interventional and random allocation was possible, was it described? |  |  |  | x |
| 6 | If interventional and blinding of investigators was possible, was it reported? |  |  |  | x |
| 7 | If interventional and blinding of subjects was possible, was it reported? |  |  |  | x |
| 8 | Outcome and (if applicable) exposure measure(s) well defined and robust to measurement / misclassification bias? Means of assessment reported? |  | x |  |  |
| 9 | Sample size appropriate? | x |  |  |  |
| 10 | Analytic methods described/justified and appropriate? | x |  |  |  |
| 11 | Some estimate of variance is reported for the main results? | x |  |  |  |
| 12 | Controlled for confounding? |  |  |  | x |
| 13 | Results reported in sufficient detail? | x |  |  |  |
| 14 | Conclusions supported by the results? | x |  |  |  |

**Total score: 19 – (4 x 2) = 15/16**

**[74]** Miller, M. & Neaigus, A. (2002). Sex partner support, drug use and sex risk among HIV-negative non-injecting users. *AIDS Care, 14* (6), 801-813.

|  | **Criteria** | **Yes**  **(2)** | **Partial**  **(1)** | **No**  **(0)** | **N/A** |
| --- | --- | --- | --- | --- | --- |
| 1 | Question / objective sufficiently described? |  | x |  |  |
| 2 | Study design evident and appropriate? |  | x |  |  |
| 3 | Method of subject/comparison group selection or source of information/input variables described and appropriate? | x |  |  |  |
| 4 | Subject (and comparison group, if applicable) characteristics sufficiently described? | x |  |  |  |
| 5 | If interventional and random allocation was possible, was it described? |  |  |  | x |
| 6 | If interventional and blinding of investigators was possible, was it reported? |  |  |  | x |
| 7 | If interventional and blinding of subjects was possible, was it reported? |  |  |  | x |
| 8 | Outcome and (if applicable) exposure measure(s) well defined and robust to measurement / misclassification bias? Means of assessment reported? | x |  |  |  |
| 9 | Sample size appropriate? | x |  |  |  |
| 10 | Analytic methods described/justified and appropriate? | x |  |  |  |
| 11 | Some estimate of variance is reported for the main results? | x |  |  |  |
| 12 | Controlled for confounding? |  |  |  | x |
| 13 | Results reported in sufficient detail? | x |  |  |  |
| 14 | Conclusions supported by the results? | x |  |  |  |

**Total score: 18 – (4 x 2) = 14/16**

**[76]** Sanchez, J., Comerford, M., Chitwood, D. D., Fernandez, M. I. & McCoy, C. B. (2002). High risk sexual behaviours among heroin sniffers who have no history of injection drug use: Implications for HIV risk reduction. *AIDS Care, 14* (3), 391-398.

|  | **Criteria** | **Yes**  **(2)** | **Partial**  **(1)** | **No**  **(0)** | **N/A** |
| --- | --- | --- | --- | --- | --- |
| 1 | Question / objective sufficiently described? |  | x |  |  |
| 2 | Study design evident and appropriate? | x |  |  |  |
| 3 | Method of subject/comparison group selection or source of information/input variables described and appropriate? | x |  |  |  |
| 4 | Subject (and comparison group, if applicable) characteristics sufficiently described? | x |  |  |  |
| 5 | If interventional and random allocation was possible, was it described? |  |  |  | x |
| 6 | If interventional and blinding of investigators was possible, was it reported? |  |  |  | x |
| 7 | If interventional and blinding of subjects was possible, was it reported? |  |  |  | x |
| 8 | Outcome and (if applicable) exposure measure(s) well defined and robust to measurement / misclassification bias? Means of assessment reported? |  | x |  |  |
| 9 | Sample size appropriate? | x |  |  |  |
| 10 | Analytic methods described/justified and appropriate? |  | x |  |  |
| 11 | Some estimate of variance is reported for the main results? | x |  |  |  |
| 12 | Controlled for confounding? |  |  |  | x |
| 13 | Results reported in sufficient detail? | x |  |  |  |
| 14 | Conclusions supported by the results? | x |  |  |  |

**Total score: 17 – (4 x 2) = 9/16**

**[49]** Tyndall, M. W., Patrick, D., Spittal, P., Li, K., O’Shaughnessy, M. V. & Schechter, M. T. (2002). *Sexually Transmitted Infections, 78* (S1), i170-i175.

|  | **Criteria** | **Yes**  **(2)** | **Partial**  **(1)** | **No**  **(0)** | **N/A** |
| --- | --- | --- | --- | --- | --- |
| 1 | Question / objective sufficiently described? |  | x |  |  |
| 2 | Study design evident and appropriate? | x |  |  |  |
| 3 | Method of subject/comparison group selection or source of information/input variables described and appropriate? |  | x |  |  |
| 4 | Subject (and comparison group, if applicable) characteristics sufficiently described? |  | x |  |  |
| 5 | If interventional and random allocation was possible, was it described? |  |  |  | x |
| 6 | If interventional and blinding of investigators was possible, was it reported? |  |  |  | x |
| 7 | If interventional and blinding of subjects was possible, was it reported? |  |  |  | x |
| 8 | Outcome and (if applicable) exposure measure(s) well defined and robust to measurement / misclassification bias? Means of assessment reported? |  | x |  |  |
| 9 | Sample size appropriate? | x |  |  |  |
| 10 | Analytic methods described/justified and appropriate? | x |  |  |  |
| 11 | Some estimate of variance is reported for the main results? | x |  |  |  |
| 12 | Controlled for confounding? |  |  |  | x |
| 13 | Results reported in sufficient detail? | x |  |  |  |
| 14 | Conclusions supported by the results? | x |  |  |  |

**Total score: 16 – (4 x 2) = 8/16**

**[66]** Gore-Felton, C., Somlai, A. M., Benotsch, E. G., Kelly, J. A., Ostrovski, D. & Kozlov, A. (2003). The influence of gender on factors associated with HIV transmission risk among young Russian injection drug users. *The American Journal of Drug and Alcohol Abuse, 29* (4), 881-894.

|  | **Criteria** | **Yes**  **(2)** | **Partial**  **(1)** | **No**  **(0)** | **N/A** |
| --- | --- | --- | --- | --- | --- |
| 1 | Question / objective sufficiently described? |  |  | x |  |
| 2 | Study design evident and appropriate? |  |  |  |  |
| 3 | Method of subject/comparison group selection or source of information/input variables described and appropriate? | x |  |  |  |
| 4 | Subject (and comparison group, if applicable) characteristics sufficiently described? | x |  |  |  |
| 5 | If interventional and random allocation was possible, was it described? |  |  |  | x |
| 6 | If interventional and blinding of investigators was possible, was it reported? |  |  |  | x |
| 7 | If interventional and blinding of subjects was possible, was it reported? |  |  |  | x |
| 8 | Outcome and (if applicable) exposure measure(s) well defined and robust to measurement / misclassification bias? Means of assessment reported? | x |  |  |  |
| 9 | Sample size appropriate? | x |  |  |  |
| 10 | Analytic methods described/justified and appropriate? |  |  | x |  |
| 11 | Some estimate of variance is reported for the main results? |  | x |  |  |
| 12 | Controlled for confounding? |  |  |  | x |
| 13 | Results reported in sufficient detail? |  | x |  |  |
| 14 | Conclusions supported by the results? | x |  |  |  |

**Total score: 12 – (4 x 2) = 4/16**

**[75]** Medrano, M. A., Hatch, J.P., Zule, W. A. & Desmond, D. P. (2003). Childhood trauma and adult prostitution behavior in a multi-ethnic heterosexual drug using population. *The American Journal of Drug and Alcohol Abuse, 29* (2), 463-486.

|  | **Criteria** | **Yes**  **(2)** | **Partial**  **(1)** | **No**  **(0)** | **N/A** |
| --- | --- | --- | --- | --- | --- |
| 1 | Question / objective sufficiently described? |  | x |  |  |
| 2 | Study design evident and appropriate? | x |  |  |  |
| 3 | Method of subject/comparison group selection or source of information/input variables described and appropriate? | x |  |  |  |
| 4 | Subject (and comparison group, if applicable) characteristics sufficiently described? | x |  |  |  |
| 5 | If interventional and random allocation was possible, was it described? |  |  |  | x |
| 6 | If interventional and blinding of investigators was possible, was it reported? |  |  |  | x |
| 7 | If interventional and blinding of subjects was possible, was it reported? |  |  |  | x |
| 8 | Outcome and (if applicable) exposure measure(s) well defined and robust to measurement / misclassification bias? Means of assessment reported? | x |  |  |  |
| 9 | Sample size appropriate? | x |  |  |  |
| 10 | Analytic methods described/justified and appropriate? | x |  |  |  |
| 11 | Some estimate of variance is reported for the main results? | x |  |  |  |
| 12 | Controlled for confounding? |  |  |  | x |
| 13 | Results reported in sufficient detail? | x |  |  |  |
| 14 | Conclusions supported by the results? | x |  |  |  |

**Total score: 19 – (4 x 2) = 15/16**

**[67]** Wang, Q. & Lin, G. (2003). Sex exchange and HIV-related risk behaviors among female heroin users in China. *The Journal of Drug Issues,* 119-132.

|  | **Criteria** | **Yes**  **(2)** | **Partial**  **(1)** | **No**  **(0)** | **N/A** |
| --- | --- | --- | --- | --- | --- |
| 1 | Question / objective sufficiently described? |  | x |  |  |
| 2 | Study design evident and appropriate? | x |  |  |  |
| 3 | Method of subject/comparison group selection or source of information/input variables described and appropriate? |  |  | x |  |
| 4 | Subject (and comparison group, if applicable) characteristics sufficiently described? | x |  |  |  |
| 5 | If interventional and random allocation was possible, was it described? |  |  |  | x |
| 6 | If interventional and blinding of investigators was possible, was it reported? |  |  |  | x |
| 7 | If interventional and blinding of subjects was possible, was it reported? |  |  |  | x |
| 8 | Outcome and (if applicable) exposure measure(s) well defined and robust to measurement / misclassification bias? Means of assessment reported? | x |  |  |  |
| 9 | Sample size appropriate? | x |  |  |  |
| 10 | Analytic methods described/justified and appropriate? | x |  |  |  |
| 11 | Some estimate of variance is reported for the main results? | x |  |  |  |
| 12 | Controlled for confounding? |  |  |  | x |
| 13 | Results reported in sufficient detail? | x |  |  |  |
| 14 | Conclusions supported by the results? | x |  |  |  |

**Total score: 17 – (4 x 2) = 9/16**

**[68]** Bell, A. V., Ompad, D. & Sherman, S. G. (2006). Sexual and drug risk behaviors among women who have sex with women. *American Journal of Public Health, 96* (6), 1066-1072.

|  | **Criteria** | **Yes**  **(2)** | **Partial**  **(1)** | **No**  **(0)** | **N/A** |
| --- | --- | --- | --- | --- | --- |
| 1 | Question / objective sufficiently described? | x |  |  |  |
| 2 | Study design evident and appropriate? | x |  |  |  |
| 3 | Method of subject/comparison group selection or source of information/input variables described and appropriate? | x |  |  |  |
| 4 | Subject (and comparison group, if applicable) characteristics sufficiently described? | x |  |  |  |
| 5 | If interventional and random allocation was possible, was it described? |  |  |  | x |
| 6 | If interventional and blinding of investigators was possible, was it reported? |  |  |  | x |
| 7 | If interventional and blinding of subjects was possible, was it reported? |  |  |  | x |
| 8 | Outcome and (if applicable) exposure measure(s) well defined and robust to measurement / misclassification bias? Means of assessment reported? | x |  |  |  |
| 9 | Sample size appropriate? | x |  |  |  |
| 10 | Analytic methods described/justified and appropriate? | x |  |  |  |
| 11 | Some estimate of variance is reported for the main results? | x |  |  |  |
| 12 | Controlled for confounding? |  |  |  | x |
| 13 | Results reported in sufficient detail? | x |  |  |  |
| 14 | Conclusions supported by the results? | x |  |  |  |

**Total score: 20 – (4 x 2) = 16/16**

**[51]** Gu, J., Chen, H., Chen, X., Lau, J. T. F., Wang, R., Liu, C., Liu, J., Lei, Z. & Li, Z. (2008). Severity of drug dependence, economic pressure and HIV-related risk behaviors among non-institutionalized female injecting drug users who are also sex workers in China. *Drug and Alcohol Dependence, 97*, 257-267.

|  | **Criteria** | **Yes**  **(2)** | **Partial**  **(1)** | **No**  **(0)** | **N/A** |
| --- | --- | --- | --- | --- | --- |
| 1 | Question / objective sufficiently described? |  | x |  |  |
| 2 | Study design evident and appropriate? | x |  |  |  |
| 3 | Method of subject/comparison group selection or source of information/input variables described and appropriate? | x |  |  |  |
| 4 | Subject (and comparison group, if applicable) characteristics sufficiently described? | x |  |  |  |
| 5 | If interventional and random allocation was possible, was it described? |  |  |  | x |
| 6 | If interventional and blinding of investigators was possible, was it reported? |  |  |  | x |
| 7 | If interventional and blinding of subjects was possible, was it reported? |  |  |  | x |
| 8 | Outcome and (if applicable) exposure measure(s) well defined and robust to measurement / misclassification bias? Means of assessment reported? | x |  |  |  |
| 9 | Sample size appropriate? | x |  |  |  |
| 10 | Analytic methods described/justified and appropriate? | x |  |  |  |
| 11 | Some estimate of variance is reported for the main results? | x |  |  |  |
| 12 | Controlled for confounding? |  |  |  | x |
| 13 | Results reported in sufficient detail? | x |  |  |  |
| 14 | Conclusions supported by the results? | x |  |  |  |

**Total score: 19 – (4 x 2) = 15/16**

**[78]** Gu, J., Wang, R., Chen, H., Lau, J. T. F., Zhang, L., Hu, X., Lei, Z., Li, Z., Cai, H., Wang, T. & Tsui, H. (2009). Prevalence of needle sharing, commercial sex behaviors and associated factors in Chinese male and female injecting drug user populations. *AIDS Care, 21* (1), 31-41.

|  | **Criteria** | **Yes**  **(2)** | **Partial**  **(1)** | **No**  **(0)** | **N/A** |
| --- | --- | --- | --- | --- | --- |
| 1 | Question / objective sufficiently described? | x |  |  |  |
| 2 | Study design evident and appropriate? | x |  |  |  |
| 3 | Method of subject/comparison group selection or source of information/input variables described and appropriate? | x |  |  |  |
| 4 | Subject (and comparison group, if applicable) characteristics sufficiently described? | x |  |  |  |
| 5 | If interventional and random allocation was possible, was it described? |  |  |  | x |
| 6 | If interventional and blinding of investigators was possible, was it reported? |  |  |  | x |
| 7 | If interventional and blinding of subjects was possible, was it reported? |  |  |  | x |
| 8 | Outcome and (if applicable) exposure measure(s) well defined and robust to measurement / misclassification bias? Means of assessment reported? | x |  |  |  |
| 9 | Sample size appropriate? | x |  |  |  |
| 10 | Analytic methods described/justified and appropriate? | x |  |  |  |
| 11 | Some estimate of variance is reported for the main results? | x |  |  |  |
| 12 | Controlled for confounding? |  |  |  | x |
| 13 | Results reported in sufficient detail? | x |  |  |  |
| 14 | Conclusions supported by the results? | x |  |  |  |

**Total score: 20 – (4 x 2) = 16/16**

**[69]** Cavanaugh, C. E., Floyd, L. J., Penniman, T. V., Hulbert, A., Gaydos, C. & Latimer, W. W. (2011). Examining racial/ethnic disparities in sexually transmitted diseases among recent heroin-using and cocaine-using women. *Journal of Women’s Health, 20* (2), 197-205.

|  | **Criteria** | **Yes**  **(2)** | **Partial**  **(1)** | **No**  **(0)** | **N/A** |
| --- | --- | --- | --- | --- | --- |
| 1 | Question / objective sufficiently described? | x |  |  |  |
| 2 | Study design evident and appropriate? | x |  |  |  |
| 3 | Method of subject/comparison group selection or source of information/input variables described and appropriate? | x |  |  |  |
| 4 | Subject (and comparison group, if applicable) characteristics sufficiently described? | x |  |  |  |
| 5 | If interventional and random allocation was possible, was it described? |  |  |  | x |
| 6 | If interventional and blinding of investigators was possible, was it reported? |  |  |  | x |
| 7 | If interventional and blinding of subjects was possible, was it reported? |  |  |  | x |
| 8 | Outcome and (if applicable) exposure measure(s) well defined and robust to measurement / misclassification bias? Means of assessment reported? | x |  |  |  |
| 9 | Sample size appropriate? | x |  |  |  |
| 10 | Analytic methods described/justified and appropriate? | x |  |  |  |
| 11 | Some estimate of variance is reported for the main results? | x |  |  |  |
| 12 | Controlled for confounding? |  |  |  | x |
| 13 | Results reported in sufficient detail? | x |  |  |  |
| 14 | Conclusions supported by the results? | x |  |  |  |

**Total score: 20 – (4 x 2) = 16/16**

**[70]** Peng, E. Y-C., Yeh, C-Y., Cheng, S-H., Morisky, D. E., Lan, Y-C., Chen, Y-M. A., Lyu, S-Y. & Malow, R. M. (2011). A case-control study of HIV infection among incarcerated female drug users: Impact of sharing needles and having drug-using sexual partners. *Journal of the Formosan Medical Association, 110* (7), 446-453.

|  | **Criteria** | **Yes**  **(2)** | **Partial**  **(1)** | **No**  **(0)** | **N/A** |
| --- | --- | --- | --- | --- | --- |
| 1 | Question / objective sufficiently described? |  | x |  |  |
| 2 | Study design evident and appropriate? | x |  |  |  |
| 3 | Method of subject/comparison group selection or source of information/input variables described and appropriate? |  | x |  |  |
| 4 | Subject (and comparison group, if applicable) characteristics sufficiently described? | x |  |  |  |
| 5 | If interventional and random allocation was possible, was it described? |  |  |  | x |
| 6 | If interventional and blinding of investigators was possible, was it reported? |  |  |  | x |
| 7 | If interventional and blinding of subjects was possible, was it reported? |  |  |  | x |
| 8 | Outcome and (if applicable) exposure measure(s) well defined and robust to measurement / misclassification bias? Means of assessment reported? | x |  |  |  |
| 9 | Sample size appropriate? | x |  |  |  |
| 10 | Analytic methods described/justified and appropriate? | x |  |  |  |
| 11 | Some estimate of variance is reported for the main results? | x |  |  |  |
| 12 | Controlled for confounding? |  |  |  | x |
| 13 | Results reported in sufficient detail? |  | x |  |  |
| 14 | Conclusions supported by the results? | x |  |  |  |

**Total score: 17 – (4 x 2) = 9/16**

**[52]** Gaines, T. L., Rudolph, A. E., Brouwer, K. C., Strathdee, S. A., Lozada, R., Martinez, G., Goldenberg, S. M. & Rusch, M. L. A. (2013). The longitudinal association of venue stability with consistent condom use among female sex workers in two Mexico-USA border cities. *International Journal of STD & AIDS, 24* (7), 523-529.

|  | **Criteria** | **Yes**  **(2)** | **Partial**  **(1)** | **No**  **(0)** | **N/A** |
| --- | --- | --- | --- | --- | --- |
| 1 | Question / objective sufficiently described? | x |  |  |  |
| 2 | Study design evident and appropriate? | x |  |  |  |
| 3 | Method of subject/comparison group selection or source of information/input variables described and appropriate? |  | x |  |  |
| 4 | Subject (and comparison group, if applicable) characteristics sufficiently described? | x |  |  |  |
| 5 | If interventional and random allocation was possible, was it described? |  |  |  | x |
| 6 | If interventional and blinding of investigators was possible, was it reported? |  |  |  | x |
| 7 | If interventional and blinding of subjects was possible, was it reported? |  |  |  | x |
| 8 | Outcome and (if applicable) exposure measure(s) well defined and robust to measurement / misclassification bias? Means of assessment reported? | x |  |  |  |
| 9 | Sample size appropriate? | x |  |  |  |
| 10 | Analytic methods described/justified and appropriate? | x |  |  |  |
| 11 | Some estimate of variance is reported for the main results? | x |  |  |  |
| 12 | Controlled for confounding? |  |  |  | x |
| 13 | Results reported in sufficient detail? | x |  |  |  |
| 14 | Conclusions supported by the results? | x |  |  |  |

**Total score: 19 – (4 x 2) = 15/16**

**[71]** Goldenberg, S. M., Rangel, G., Staines, H., Vera, A., Lozada, R., Nguyen, L., Silverman, J. G. & Strathdee, S. A. (2013). Individual, interpersonal, and social-structural correlated of involuntary sex exchange among female sex workers in two Mexico-U.S. border cities. *Journal of Acquired Immune Deficiency Syndromes, 63* (5), 639-646.

|  | **Criteria** | **Yes**  **(2)** | **Partial**  **(1)** | **No**  **(0)** | **N/A** |
| --- | --- | --- | --- | --- | --- |
| 1 | Question / objective sufficiently described? |  | x |  |  |
| 2 | Study design evident and appropriate? | x |  |  |  |
| 3 | Method of subject/comparison group selection or source of information/input variables described and appropriate? | x |  |  |  |
| 4 | Subject (and comparison group, if applicable) characteristics sufficiently described? | x |  |  |  |
| 5 | If interventional and random allocation was possible, was it described? |  |  |  | x |
| 6 | If interventional and blinding of investigators was possible, was it reported? |  |  |  | x |
| 7 | If interventional and blinding of subjects was possible, was it reported? |  |  |  | x |
| 8 | Outcome and (if applicable) exposure measure(s) well defined and robust to measurement / misclassification bias? Means of assessment reported? | x |  |  |  |
| 9 | Sample size appropriate? | x |  |  |  |
| 10 | Analytic methods described/justified and appropriate? | x |  |  |  |
| 11 | Some estimate of variance is reported for the main results? | x |  |  |  |
| 12 | Controlled for confounding? |  |  |  | x |
| 13 | Results reported in sufficient detail? | x |  |  |  |
| 14 | Conclusions supported by the results? | x |  |  |  |

**Total score: 19 – (4 x 2) = 15/16**

**[50]** Mackesy-Amiti, M. E., Noodram, B., Williams, C., Ouellet, L. J. & Broz, D. (2013). Sexual risk behaviour associated with transition to injection among young non-injecting heroin users. *AIDS and Behavior, 17*, 2459-2466.

|  | **Criteria** | **Yes**  **(2)** | **Partial**  **(1)** | **No**  **(0)** | **N/A** |
| --- | --- | --- | --- | --- | --- |
| 1 | Question / objective sufficiently described? |  | x |  |  |
| 2 | Study design evident and appropriate? | x |  |  |  |
| 3 | Method of subject/comparison group selection or source of information/input variables described and appropriate? | x |  |  |  |
| 4 | Subject (and comparison group, if applicable) characteristics sufficiently described? | x |  |  |  |
| 5 | If interventional and random allocation was possible, was it described? |  |  |  | x |
| 6 | If interventional and blinding of investigators was possible, was it reported? |  |  |  | x |
| 7 | If interventional and blinding of subjects was possible, was it reported? |  |  |  | x |
| 8 | Outcome and (if applicable) exposure measure(s) well defined and robust to measurement / misclassification bias? Means of assessment reported? | x |  |  |  |
| 9 | Sample size appropriate? | x |  |  |  |
| 10 | Analytic methods described/justified and appropriate? | x |  |  |  |
| 11 | Some estimate of variance is reported for the main results? | x |  |  |  |
| 12 | Controlled for confounding? |  |  |  | x |
| 13 | Results reported in sufficient detail? | x |  |  |  |
| 14 | Conclusions supported by the results? | x |  |  |  |

**Total score: 19 – (4 x 2) = 15/16**

**[72]** Iversen, J., Dolan, K., Ezard, N. & Maher, L. (2015). HIV and Hepatitis C virus infection and risk behaviors among heterosexual, bisexual, and lesbian women who inject drugs in Australia. *LGTB Health, 2* (2), 127-134.

|  | **Criteria** | **Yes**  **(2)** | **Partial**  **(1)** | **No**  **(0)** | **N/A** |
| --- | --- | --- | --- | --- | --- |
| 1 | Question / objective sufficiently described? |  | x |  |  |
| 2 | Study design evident and appropriate? | x |  |  |  |
| 3 | Method of subject/comparison group selection or source of information/input variables described and appropriate? | x |  |  |  |
| 4 | Subject (and comparison group, if applicable) characteristics sufficiently described? | x |  |  |  |
| 5 | If interventional and random allocation was possible, was it described? |  |  |  | x |
| 6 | If interventional and blinding of investigators was possible, was it reported? |  |  |  | x |
| 7 | If interventional and blinding of subjects was possible, was it reported? |  |  |  | x |
| 8 | Outcome and (if applicable) exposure measure(s) well defined and robust to measurement / misclassification bias? Means of assessment reported? | x |  |  |  |
| 9 | Sample size appropriate? | x |  |  |  |
| 10 | Analytic methods described/justified and appropriate? |  | x |  |  |
| 11 | Some estimate of variance is reported for the main results? | x |  |  |  |
| 12 | Controlled for confounding? |  |  |  | x |
| 13 | Results reported in sufficient detail? | x |  |  |  |
| 14 | Conclusions supported by the results? | x |  |  |  |

**Total score: 18 – (4 x 2) = 14/16**

**(2)**

**[77]** Syvertsen, J. L., Robertson Bazzi, A., Martinez, G., Rangel, G., Ulibarri, M. D., Fergus, K. B., Amaro, H. & Strathdee, S. A. (2015). Love, trust, and HIV risk among female sex workers and their intimate male partners. *American Journal of Public Health, 105* (8), 1667-1674.**TIAL**

|  | **Criteria** | **Yes**  **(2)** | **Partial**  **(1)** | **No**  **(0)** | **N/A** |
| --- | --- | --- | --- | --- | --- |
| 1 | Question / objective sufficiently described? | x |  |  |  |
| 2 | Study design evident and appropriate? | x |  |  |  |
| 3 | Method of subject/comparison group selection or source of information/input variables described and appropriate? | x |  |  |  |
| 4 | Subject (and comparison group, if applicable) characteristics sufficiently described? | x |  |  |  |
| 5 | If interventional and random allocation was possible, was it described? |  |  |  | x |
| 6 | If interventional and blinding of investigators was possible, was it reported? |  |  |  | x |
| 7 | If interventional and blinding of subjects was possible, was it reported? |  |  |  | x |
| 8 | Outcome and (if applicable) exposure measure(s) well defined and robust to measurement / misclassification bias? Means of assessment reported? | x |  |  |  |
| 9 | Sample size appropriate? | x |  |  |  |
| 10 | Analytic methods described/justified and appropriate? | x |  |  |  |
| 11 | Some estimate of variance is reported for the main results? | x |  |  |  |
| 12 | Controlled for confounding? |  |  |  | x |
| 13 | Results reported in sufficient detail? | x |  |  |  |
| 14 | Conclusions supported by the results? | x |  |  |  |

**Total score: 20 – (4 x 2) = 16/16**
